# The relativistic Euler equations with a physical vacuum boundary: Hadamard local well-posedness, rough solutions, and continuation criterion

**DOI:** 10.1007/s00205-022-01783-3

**Published:** 2022-05-10

**Authors:** Marcelo M. Disconzi, Mihaela Ifrim, Daniel Tataru

**Affiliations:** 1 Vanderbilt University, Nashville, TN USA; 2 Department of Mathematics, University of Wisconsin, Madison, USA; 3 Department of Mathematics, University of California at Berkeley, Berkeley, USA

## Abstract

In this paper we provide a complete local well-posedness theory for the free boundary relativistic Euler equations with a physical vacuum boundary on a Minkowski background. Specifically, we establish the following results: (i) local well-posedness in the Hadamard sense, i.e., local existence, uniqueness, and continuous dependence on the data; (ii) low regularity solutions: our uniqueness result holds at the level of Lipschitz velocity and density, while our rough solutions, obtained as unique limits of smooth solutions, have regularity only a half derivative above scaling; (iii) stability: our uniqueness in fact follows from a more general result, namely, we show that a certain nonlinear functional that tracks the distance between two solutions (in part by measuring the distance between their respective boundaries) is propagated by the flow; (iv) we establish sharp, essentially scale invariant energy estimates for solutions; (v) a sharp continuation criterion, at the level of scaling, showing that solutions can be continued as long as the velocity is in $L^1_t Lip$ and a suitable weighted version of the density is at the same regularity level. Our entire approach is in Eulerian coordinates and relies on the functional framework developed in the companion work of the second and third authors on corresponding non relativistic problem. All our results are valid for a general equation of state $p(\varrho )= \varrho ^\gamma$, $\gamma > 1$.

## Introduction

In this article, we consider the relativistic Euler equations, which describe the motion of a relativistic fluid in a Minkowski background $\mathbb M^{d+1}$, $d \ge 1$. The fluid state is represented by the (energy) *density* $\varrho \ge 0$, and the *relativistic velocity* *u*. The velocity is assumed to be a forward time-like vector field, normalized by

1.1 $$\begin{aligned} u^\alpha u_\alpha = -1. \end{aligned}$$

The equations of motion consist of

1.2 $$\begin{aligned} \partial _\alpha \mathcal {T}^\alpha _\beta = 0, \end{aligned}$$

where $\mathcal {T}$ is the energy-momentum tensor for a perfect fluid, defined by

1.3 $$\begin{aligned} \mathcal {T}_{\alpha \beta } {:=} (p + \varrho ) u_\alpha u_\beta + p \, m_{\alpha \beta }. \end{aligned}$$

Here $m$ is the Minkowski metric, and *p* is the *pressure*, which is subject to the equation of state

$$\begin{aligned} p = p(\varrho ) . \end{aligned}$$

Projecting (1.2) onto the directions parallel and perpendicular to *u*, using definition (1.3), and the identity (1.1), yields the system

1.4 $$\begin{aligned} \left\{ \begin{aligned}&u^\mu \partial _\mu \varrho + (p + \varrho )\partial _\mu u^\mu = 0\\&(p+\varrho ) u^\mu \partial _\mu u_\alpha + \Pi ^\mu _\alpha \partial _\mu p = 0, \end{aligned}\right. \end{aligned}$$

with *u* satisfying the constraint (1.1), which is in turn preserved by the time evolution. Here $\Pi$ is is the projection on the space orthogonal to *u* and is given by

$$\begin{aligned} \Pi _{\alpha \beta } = m_{\alpha \beta } + u_\alpha u_\beta . \end{aligned}$$

Throughout this paper, we adopt standard rectangular coordinates in Minkowski space, denoted by $\{x^0,x^1,\dots ,x^d\}$, and we identify $x^0$ with a time coordinate, $t{:=} x^0$. Greek indices vary from 0 to *d* and Latin indices from 1 to *d*.

The system (1.4) can be seen as a nonlinear hyperbolic system, which in the reference frame of the moving fluid has the propagation speed

$$\begin{aligned} c_s^2(\varrho ) {:=} \frac{ d p }{d\varrho }, \end{aligned}$$

which is subject to

$$\begin{aligned} 0 \le c_s < 1, \end{aligned}$$

implying that the speed of propagation of sound waves is always non-negative and below the speed of light (which equals to one in the units we adopted).

In this article we consider the physical situation where vacuum states are allowed, i.e. the density is allowed to vanish. The gas is located in the moving domain

$$\begin{aligned} \Omega _t{:=} \{ x\in \mathbb {R}^{d} \, | \, \varrho (t,x) > 0 \}, \end{aligned}$$

whose boundary $\Gamma _t$ is the *vacuum boundary*, which is advected by the fluid velocity *u*.

The distinguishing characteristic of a gas, versus the case of a liquid, is that the density, and implicitly the pressure and the sound speed, vanish on the free boundary $\Gamma _t$,

$$\begin{aligned} \varrho = 0, \quad p = 0, \quad c_s = 0 \qquad \text { on } \Gamma _t. \end{aligned}$$

Thus, the equations studied here provide a basic model of relativistic gaseous stars (see Section 1.6). An appropriate equation of state to describe this situation is^1^ (see,e.g., [37], Section 2.4] or [35]):

1.5 $$\begin{aligned} p(\varrho ) = \varrho ^{\kappa +1}, \, \text { where } \kappa > 0 \text { is a constant.} \end{aligned}$$

The decay rate of the sound speed at the free boundary plays a critical role. Precisely, there is a unique, natural decay rate which is consistent with the time evolution of the free boundary problem for the relativistic Euler gas, which is commonly referred to as *physical vacuum*, and has the form

1.6 $$\begin{aligned} c^2_s (t,x) \approx {\text {dist}}(x,\Gamma _t) \qquad \text { in } \Omega _t, \end{aligned}$$

where ${\text {dist}} (\cdot , \cdot )$ is the distance function. Exactly the same requirement is present in the non-relativistic compressible Euler equations. As in the non-relativistic setting, (1.6) should be considered as a condition on the initial data that is propagated by the time-evolution.

There are two classical approaches in fluid dynamics, using either Eulerian coordinates, where the reference frame is fixed and the fluid particles are moving, or using Lagrangian coordinates, where the particles are stationary but the frame is moving. Both of these approaches have been extensively developed in the context of the Euler equations, where the local well-posedness problem is very well understood.

By contrast, the free boundary problem corresponding to the physical vacuum has been far less studied and understood. Because of the difficulties related to the need to track the evolution of the free boundary, all the prior work is in the Lagrangian setting and in high regularity spaces which are only indirectly defined.

Our goal in this paper is to provide the *first local well-posedness result for this problem.* Unlike previous approaches, which were limited to proving energy-type estimates at high regularity and in a Lagrangian setting [12, 16], here we consider this problem fully within the Eulerian framework, where we provide a complete local well-posedness theory, in the Hadamard sense, in a low regularity setting. We summarize here the main features of our result, which mirror the results in the last two authors’ prior paper devoted to the non-relativistic problem [14], referring to Section 1.5 for precise statements:

- We prove the *uniqueness* of solutions with very limited regularity $v \in Lip$, $\varrho \in Lip$^2^. More generally, at the same regularity level we prove *stability*, by showing that bounds for a certain nonlinear distance between different solutions can be propagated in time.
- Inspired by [14], we set up the Eulerian Sobolev *function space structure* where this problem should be considered, providing the correct, natural scale of spaces for this evolution.
- We prove sharp, *scale invariant*^3^*energy estimates* within the above mentioned scale of spaces, which guarantee that the appropriate Sobolev regularity of solutions can be continued for as long as we have uniform bounds at the same scale $v \in Lip$.
- We give a constructive proof of *existence* for regular solutions, fully within the Eulerian setting, based on the above energy estimates.
- We employ a nonlinear Littlewood-Paley type method, developed prior work [14], in order to obtain *rough solutions* as unique limits of smooth solutions. This also yields the *continuous dependence* of the solutions on the initial data.

### Space-time foliations and the material derivative

The relativistic character of our problem implies that there is no preferred choice of coordinates. On the other hand, in order to derive estimates and make quantitative assertions about the evolution, we have to choose a foliation of spacetime by space-like hypersurfaces. Here, we take advantage of the natural set-up provided by Minkowski space and foliate the spacetime by $\{ t = constant \}$ slices. We then define the material derivative, which is adapted to this specific foliation, as

1.7 $$\begin{aligned} D_{t}:= \partial _t + \frac{u^i}{u^0} \partial _i. \end{aligned}$$

The vectorfield $D_{t}$ is better adapted to the study of the free-boundary evolution than working directly with $u^\mu \partial _\mu$. Indeed, in order to track the motion of fluid particles on the boundary, we need to understand their velocity relative to the aforementioned spacetime foliation. The velocity that is measured by an observer in a reference frame characterized by the coordinates $(t,x^1,\dots ,x^d)$ is $u^i/u^0$. This is a consequence of the fact that in relativity observers are defined by their world-lines, which can be reparametrized. This ambiguity is fixed by imposing the constraint $u^\mu u_\mu = -1$. As a consequence, the *d*-dimensional vectorfield $(u^1,\dots ,u^d)$ can have norm arbitrarily large, while the physical velocity has to have norm at most one (the speed of light).

It follows, in particular, that fluid particles on the boundary move with velocity $u^i/u^0$. These considerations also imply that the standard differentiation formula for moving domains holds with $D_{t}$, i.e.,

1.8 $$\begin{aligned} \frac{d}{dt} \int _{\Omega _t} f \, dx = \int _{\Omega _t} D_{t}f \, dx+ \int _{ \Omega _t} f \partial _i \left( \frac{u^i}{u^0} \right) \, dx. \end{aligned}$$

This formula remains valid with the good variable *v* we introduce below since $v^i/v^0 = u^i/u^0$.

### The good variables

The starting point of our analysis is a good choice of dynamical variables. We seek variables that are tailored to the characteristics of the Euler flow *all the way to moving boundary,* where the sound characteristics degenerate due to the vanishing of the sound speed. Our choice of good variables will

(i) better diagonalize the system with respect to the material derivative,
(ii) be associated with truly relativistic properties of the vorticity, and
(iii) lead to good weights that allow us to control the behavior of the fluid variables when one approaches the boundary.

Property (i) will be intrinsically tied with both the wave and transport character of the flow in that (a) the diagonalized equations lead to good second order equations that capture the propagation of sound in the fluid, see Section 3.2, and (b) it provides a good transport structure that will allow us to implement a time discretization for the construction of regular solution, see Section 6. Property (ii) will ensure a good coupling between the wave-part and the transport-part of the system. Finally, property (iii) will lead to the correct functional framework needed to close the estimates.^4^ Our good variables, denoted by (*r*, *v*), are defined in (1.9) and (1.15). The corresponding equations of motion are (1.16), which we now derive.

Our first choice of good variables is a rescaled version of the velocity given by

1.9 $$\begin{aligned} v^\alpha = f(\varrho ) u^\alpha , \end{aligned}$$

where *f* is given by

1.10 $$\begin{aligned} f(\varrho ) {:=} \exp \int \frac{c_s^2(\varrho )}{p(\varrho ) + \varrho } \, d\varrho . \end{aligned}$$

Although we are interested in the case $p(\varrho ) = \varrho ^{\kappa +1}$, it is instructive to consider first a general barotropic equation of state; see the discussion related to the vorticity further below.

In order to understand our choice for *f*, compute

$$\begin{aligned} \partial _\mu v^\alpha = f^\prime (\varrho ) \partial _\mu \varrho u^\alpha + f(\varrho ) \partial _\mu u^\alpha . \end{aligned}$$

Solving for $\partial _\mu u^\alpha$ and plugging the resulting expression into the second equation of (1.4) we find

$$\begin{aligned} \frac{p +\varrho }{f} u^\mu \partial _\mu v^\alpha + c_s^2 m^{\alpha \mu } \partial _\mu \varrho + \left( -\frac{f^\prime }{f} (p + \varrho ) + c_s^2 \right) u^\alpha u^\mu \partial _\mu \varrho = 0. \end{aligned}$$

We see that the term in parenthesis vanishes if *f* is given by (1.10), resulting in an equation which is diagonal with respect to the material derivative, and which we write as

1.11 $$\begin{aligned} D_{t}v^\alpha + \frac{c_s^2 f^2}{(p+\varrho ) v^0} m^{\alpha \mu } \partial _\mu \varrho = 0. \end{aligned}$$

We notice that in terms of *v*, the material derivative (1.7) reads as

$$\begin{aligned} D_{t}= \partial _t + \frac{v^i}{v^0} \partial _i. \end{aligned}$$

In view of the constraint (1.1), we have that $v^0$ satisfies

1.12 $$\begin{aligned} v^0 = \sqrt{f^2 + |v|^2}, \quad |v|^2 {:=} v^i v_i, \end{aligned}$$

and in solving for $v^0$ we chose the positive square root because *u*, and thus *v*, is a future-pointing vectorfield.

We now show that our choice (1.9) also diagonalizes the first equation in (1.4). First, we use (1.11) with $\alpha =0$ and solve to $\partial _t v^0$, obtaining

$$\begin{aligned} \partial _{t} v^{0}&= \frac{{c_{s}^{2} f^{2} }}{{(p +{\varrho })v^{0} }}\partial _{t} {\varrho } - \frac{{v^{i} }}{{v^{0} }}\partial _{i} v^{0} \\&= \frac{{c_{s}^{2} f^{2} }}{{(p +{\varrho })v^{0} }}\partial _{t} {\varrho } - \frac{{ff^{\prime } }}{{(v^{0} )^{2} }}v^{i} \partial _{i} {\varrho } - \frac{{v^{i} v^{j} }}{{(v^{0} )^{2} }}\partial _{i} v_{j} , \end{aligned}$$

where in the second equality we used (1.12) to compute $\partial _i v^0$. Using the above identity for $\partial _t v^0$, we find the following expression for $\partial _\mu v^\mu$:

$$\begin{aligned} \partial _\mu v^\mu = \frac{c_s^2 f^2}{(p+\varrho )v^0} \partial _t \varrho - \frac{f f^\prime }{(v^0)^2} v^i \partial _i \varrho + \left( \updelta ^{ij} - \frac{v^i v^j}{(v^0)^2} \right) \partial _i v_j, \end{aligned}$$

where $\updelta$ is the Euclidean metric. Expressing $\partial _\mu u^\mu$ in terms of $\partial _\mu v^\mu$ (and derivatives of $\varrho$) and using the above expression for $\partial _\mu v^\mu$, we see that the first equation in (1.4) can be written as

1.13 $$\begin{aligned} D_{t}\varrho + \frac{p+\varrho }{ a_0 v^0} \left( \updelta ^{ij} - \frac{v^i v^j}{(v^0)^2} \right) \partial _i v_j - c_s^2 \frac{2 f^2 }{a_0 (v^0)^3} v^i \partial _i \varrho = 0. \end{aligned}$$

Here we are using the notation

1.14 $$\begin{aligned} a_0 := 1 - c_s^2\frac{|v|^2}{(v^0)^2}. \end{aligned}$$

Observe that Eqs. (1.11) and (1.13) are valid for a general barotropic equation of state. We now assume the equation of state (1.5). Then the sound speed is given by $c_s^2 = (\kappa +1)\varrho ^\kappa$ and *f* becomes $f(\varrho ) = (1+\varrho ^\kappa )^{1+\frac{1}{\kappa }}$ (we choose the constant of integration by setting $f(0)=1$, so that $v=u$ when $\varrho =0$). It turns out that it is better to adopt the sound speed squared as a primary variable instead of $\varrho$ because it plays the role of the correct weight in our energy functionals. We thus define^5^ the second component of our good variables by

1.15 $$\begin{aligned} r{:=}\frac{1+\kappa }{\kappa } \varrho ^\kappa . \end{aligned}$$

Therefore, using (*r*, *v*) as our good variables, and $p(\varrho )$ given by (1.5) we find that the Eqs. (1.11) and (1.13) become
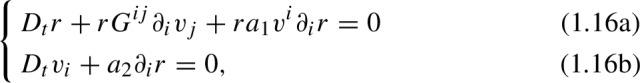


where we have defined

$$\begin{aligned} G^{ij} {:=} \frac{\kappa \langle r \rangle }{a_0 v^0} \left( \updelta ^{ij} - \frac{v^i v^j}{(v^0)^2} \right) , \quad \langle r \rangle {:=} 1+\frac{\kappa r}{\kappa +1}, \end{aligned}$$

and the coefficients $a_0,a_1$ and $a_2$ are given by

$$\begin{aligned} a_0 {:=} 1 - \kappa r \frac{|v|^2}{(v^0)^2}, \qquad a_1 {:=} -\frac{2 \kappa \langle r \rangle ^{2+\frac{2}{\kappa }} }{(v^0)^3 a_0}, \qquad a_2 {:=} \frac{\langle r \rangle ^{1+\frac{2}{\kappa }}}{v^0}. \end{aligned}$$

Equations (1.16) are the desired diagonal with respect to $D_{t}$ equations, and the rest of the article will be based on them. In writing these equations we consider only the spatial components $v^i$ as variables, with $v^0$ always given by

1.17 $$\begin{aligned} v^0 = \sqrt{\langle r \rangle ^{2+\frac{2}{\kappa }} + |v|^2}. \end{aligned}$$

The specific form of the coefficients $a_0$, $a_1$, and $a_2$ is not very important for our argument. We essentially only use that they are smooth functions of *r* and *v*, and that $a_0, \, a_2>0$.

The operator $G^{ij} \partial _i (\cdot )_j$ can be viewed as a divergence type operator. This divergence structure is related to the fact that Equations (1.16) express the wave-like behavior of *r* and of the divergence part of *v*. The symmetric and positive-definite matrix $c_s^2 G^{ij}$ is closely related to the inverse of the acoustical metric; precisely, they agree at the leading order near the boundary.

As we will see, Equations (1.16) also have the correct balance of powers of *r* to allow estimates all the way to the free boundary. The *r* factor in the divergence of *v* is related to the propagation of sound in the fluid (see Section 3.2) whereas the *r* factor in the last term of (1.16a) will allow us to treat $r a_1 v^i \partial _i r$ essentially as a perturbation at least in elliptic estimates (see Section 5).

One can always diagonalize Eq. (1.4) by simply algebraically solving for $\partial _t (\varrho ,u)$. But it is not difficult to see that this procedure will not lead to equations with good structures for the study of the vacuum boundary problem. In this regard, observe that the choice (1.9) is a nonlinear change of variables, whereas algebraically solving for $\partial _t (\varrho ,u)$ is a linear procedure.

We now comment on the relation between *v* and the vorticity of the fluid $\omega$. It is well-known (see, e.g., [5] Section IX.10.1) that in relativity the correct notion of vorticity is given by the following two-form in spacetim

1.18 $$\begin{aligned} {\begin{matrix} \omega _{\alpha \beta } {:=} (d_{st} v )_{\alpha \beta } = \partial _\alpha v_\beta - \partial _\beta v_\alpha , \end{matrix}} \end{aligned}$$

where $d_{st}$ is the exterior derivative in spacetime. This is true not only for the power law equation of state (1.5), but also for an arbitrary barotropic equation of state.

A computation using (1.18) (see, e.g., [5]) and the equations of motion implies that

1.19 $$\begin{aligned} v^\alpha \omega _{\alpha \beta } = 0, \end{aligned}$$

and that $\omega$ satisfies the following evolution equation

1.20 $$\begin{aligned} v^\mu \partial _\mu \omega _{\alpha \beta } + \partial _\alpha v^\mu \omega _{\mu \beta } + \partial _\beta v^\mu \omega _{\alpha \mu } = 0. \end{aligned}$$

Observe that (1.20) implies that $\omega = 0$ if it vanishes initially.

Since we will consider only the spatial components of *v* as independent, we use (1.19) to eliminate the 0*j* components of $\omega$ from (1.20) as follows: from (1.19) we can write

1.21 $$\begin{aligned} \omega _{0 j} = -\frac{v^i}{v^0} \omega _{i j}. \end{aligned}$$

Using (1.21) into (1.20) with $\alpha ,\beta =i,j$ we finally obtain

1.22 $$\begin{aligned} D_{t}\omega _{ij} + \frac{1}{v^0} \partial _i v^k \omega _{k j} + \frac{1}{v^0} \partial _j v^k \omega _{i k } - \frac{1}{(v^0)^2} \partial _iv^0v^k \omega _{kj} + \frac{1}{(v^0)^2} \partial _j v^0 v^k\omega _{ki} = 0. \end{aligned}$$

Equation (1.22) will be used to derive estimates for $\omega _{ij}$ that will complement the estimates for *r* and the divergence of *v* obtained from (1.16).

We remark that in the literature, the use of *v*, given by (1.9), seems to be restricted mostly to definition and evolution of the vorticity. To the best of our knowledge, this is the first time when it was observed that the same change of variables needed to define the relativistic vorticity also diagonalizes the equations of motion with respect to $D_{t}$.

### Scaling and bookkeeping scheme

Although Eq. (1.16) do not obey a scaling law, it is still possible to identify a scaling law for the leading order dynamics near the boundary. This will motivate the control norms we introduce in the next section, as well as provide a bookkeeping scheme that will allow us to streamline the analysis of many complex multilinear expressions we will encounter.

As we will see, the contribution of last term in (1.16a) to our energies is negligible, due to the multiplicative *r* factor. Thus, we ignore this term for our scaling analysis.^6^ Replacing all coefficients that are functions of (*r*, *v*) by 1, while keeping the transport and divergence structure present in the equations, we obtain the following simplified version of (1.16):
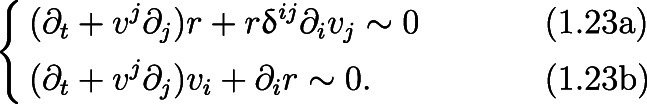


This system is expected to capture the leading order dynamics near the boundary, and also mirrors the nonrelativistic version of the compressible Euler equations, considered in the predecessor to this paper, see [14]. Equations (1.23) admit the scaling law

$$\begin{aligned} (r(t,x),v(t,x)) \mapsto (\lambda ^{-2} r(\lambda t, \lambda ^2 x), \lambda ^{-1} v(\lambda t, \lambda ^2 x) ). \end{aligned}$$

Based on this leading order scaling analysis, we assign the following order to the variables and operators in Eq. (1.16):

(i) *r* and *v* have order $-1$ and $-1/2$, respectively. More precisely, we only count *v* as having order $-1/2$ when it is differentiated. Undifferentiated *v*’s have order zero.
(ii) $D_{t}$ and $\partial _i$ have order 1/2 and 1, respectively.
(iii) $G$, $a_0$, $a_1$, and $a_2$, and more generally, any smooth function of (*r*, *v*) not vanishing at $r = 0$, have order 0.

Expanding on (iii) above, the order of a function of *r* is defined by the order of its leading term in the Taylor expansion about $r=0$, being of order zero if this leading term is a constant. The order of a multilinear expression is defined as the sum of the orders of each factor. Here we remark that all expressions arising in this paper are multilinear expressions, with the possible exception of nonlinear factors as in (iii) above.

According to this convention, all terms in equation (1.16b) have order zero, and all terms in (1.16a) have order $-1/2$, except for the last term in (1.16a) which has order $-1$. Upon successive differentiation of any multilinear expression with respect to $D_{t}$ or $\partial$, all terms produced are the same (highest) order, unless some of these derivatives apply to nonlinear factors as in (iii); then lower order terms are produced.

### Energies, function spaces, and control norms

Here we introduce the function spaces and control norms that we need in order to state our main results. A more detailed discussion is given in Section 2. With some obvious adjustments, here we follow the lat two authors’ prior work in [14]. We assume throughout that *r* is a positive function on $\Omega _t$, vanishing simply on the boundary, and so that *r* is comparable to the distance to the boundary $\Gamma _t$.

In order to identify the correct functional framework for our problem, we start with the linearization of the Eq. (1.16). In Section 3 we show that the linearized equations admit the following energy

$$\begin{aligned} \Vert (s,w)\Vert ^2_{\mathcal {H}} = \int _{\Omega _t} r^{\frac{1-\kappa }{\kappa }} ( s^2 + a_2^{-1} r G^{ij} w_i w_j )\, dx, \end{aligned}$$

which defines the (time dependent) weighted $L^2$ space ${\mathcal H }$.

The motivation for the definition of higher order norms and spaces comes from the good second order equations mentioned in Section 1.2. From Eq. (1.16), we find that the second order evolution is governed at leading order by a wave-like operator which is essentially a variable coefficient version of $D_{t}^2 - r \Delta$. This points toward higher order spaces built on powers of $r\Delta$. Taking into account also the form of the linearized energy above, we are led to the following. We define $\mathcal {H}^{2k}$ as the space of pairs of functions (*s*, *w*) in $\Omega _t$ for which the norm below is finite

$$\begin{aligned} \Vert (s,w)\Vert ^2_{\mathcal {H}^{2k}} {:=} \sum _{\vert \alpha \vert =0}^{2k} \sum _{\begin{array}{c} a=0 \\ |\alpha |- a\le k \end{array}}^{k} \Vert r^{\frac{1-\kappa }{2\kappa } + a} \partial ^\alpha s\Vert ^2_{L^2} + \sum _{\vert \alpha \vert =0}^{2k} \sum _{\begin{array}{c} a=0 \\ |\alpha |- a\le k \end{array}}^{k} \Vert r^{\frac{1-\kappa }{2\kappa }+\frac{1}{2} + a} \partial ^\alpha w \Vert ^2_{L^2}. \end{aligned}$$

The definition of $\mathcal {H}^{2k}$ for non-integer *k* is given in Section 2, via interpolation.

In view of the scaling analysis of Section 1.3, we introduce the critical space $\mathcal {H}^{2k_0}$ where

1.24 $$\begin{aligned} 2k_0 = d + 1 + \frac{1}{\kappa }, \end{aligned}$$

which has the property that its leading order homogeneous component is invariant with respect to the scaling discussed in Section 1.3. Associated with the exponent $2 k_0$ we define the following scale invariant time dependent control norm

$$\begin{aligned} A {:=} \Vert \nabla r - N \Vert _{L^\infty } + \Vert v\Vert _{\dot{C}^\frac{1}{2}}. \end{aligned}$$

here *N* is a given non-zero vectorfield with the following property. In each sufficiently small neighborhood of the boundary, there exists a $x_0 \in \Gamma _t$ such that $N(x_0) = \nabla r(x_0)$. The fact that we can choose such a *N* follows from the properties of *r*. The motivation for introducing *N* is that we can make *A* small by working in small neighborhood of each reference point $x_0$, whereas $\Vert \nabla r\Vert _{L^\infty }$ is a scale invariant quantity that cannot be made small by localization arguments.

We further introduce a second time dependent control norm that is associated with $H^{2k_0 + 1}$, given by^7^

$$\begin{aligned} B {:=} A+ \Vert \nabla r\Vert _{\tilde{C}^\frac{1}{2}} + \Vert \nabla v\Vert _{L^\infty }, \end{aligned}$$

where

$$\begin{aligned} \Vert f\Vert _{\tilde{C}^\frac{1}{2}} {:=} \sup _{x,y \in \Omega _t} \frac{ |f(x) - f(y)|}{ r(x)^\frac{1}{2} + r(y)^\frac{1}{2} + |x-y|^\frac{1}{2}}. \end{aligned}$$

It follows that $\Vert \nabla r\Vert _{\tilde{C}^\frac{1}{2}}$ scales like the $\dot{C}^\frac{3}{2}$ norm of *r*, but it is weaker in that it only uses one derivative of *r* away from the boundary. The norm *B* will control the growth of our energies, allowing for a secondary dependence on *A*.

When the density is bounded away from zero, the relativistic Euler equations can be written as a first-order symmetric hyperbolic system (see, e.g., [1]) and standard techniques can be applied to derive local estimates. The difficulties in our case come from the vanishing of *r* on the boundary. Using the finite speed of propagation of the Euler flow, we can use a partition of unity to separate the near-boundary behavior, where *r* approaches zero, from the bulk dynamics, where *r* is bounded away from zero. Furthermore, we can also localize to a small set where *A* is small. Such a localization will be implicitly assumed in all our analysis, in order to avoid cumbersome localization weights through the proofs.

### The main results

Here we state our main results. Combined, these results establish the sharp local well-posedness and continuation criterion discussed earlier. We will make all our statements for the system written in terms of the good variables (*r*, *v*), i.e., Eq. (1.16). Readers interested in the evolution of (1.4) should have no difficulty translating our statements to the original variables $\varrho$ and *u*.

We recall that Eq. (1.16) are always considered in the moving domain given by

$$\begin{aligned} \Omega {:=} \bigcup _{0\le t <T} \{ t \} \times \Omega _t, \end{aligned}$$

for some $T>0$, where the moving domain at time *t*, $\Omega _t$, is given by

$$\begin{aligned} \Omega _t= \{ x \in \mathbb {R}^d \, | \, r(t,x) > 0 \}. \end{aligned}$$

We also recall that we are interested in solutions satisfying the physical vacuum boundary condition

1.25 $$\begin{aligned} r(t,x) \approx {\text {dist}}(x,\Gamma _t), \end{aligned}$$

where ${\text {dist}} (\cdot , \cdot )$ is the distance function. Hence, by a *solution* we will always mean a pair of functions (*r*, *v*) that satisfies Eq. (1.16) within $\Omega$, and for which (1.25) holds.

We begin with our uniqueness result:

#### Theorem 1.1

(Uniqueness) Eq. (1.16) admit at most one solution (*r*, *v*) in the class

$$\begin{aligned} v \in C^1_x, \, \nabla r \in \tilde{C}^{\frac{1}{2}}_x. \end{aligned}$$

For the next Theorem, we introduce the phase space

1.26 $$\begin{aligned} \mathbf {H}^{2k} {:=} \{ (r,v) \, | \, (r,v) \in \mathcal {H}^{2k} \}. \end{aligned}$$

We refer to Section 2 for a more precise definition of $\mathbf {H}^{2k}$, including its topology. Since the $\mathcal {H}^{2k}$ norms depend on *r*, it is appropriate to think of $\mathbf {H}^{2k}$ in a nonlinear fashion, as an infinite dimensional manifold. We also stress that, while *k* was an integer in our preliminary discussion in Section 1.4, in Section 2 we extend their definition for any $k \ge 0$. Consequently, $\mathbf {H}^{2k}$ is also defined for any $k \ge 0$, and our Theorems 1.2 and 1.4 below include non-integer values of *k*.

#### Theorem 1.2

Equations (1.16) are locally well-posed in $\mathbf {H}^{2k}$ for any data $(\mathring{r},\mathring{v}) \in \mathbf {H}^{2k}$ with $\mathring{r}$ satisfying (1.25), provided that

1.27 $$\begin{aligned} 2k > 2 k_0 + 1, \end{aligned}$$

where $k_0$ is given by (1.24).

Local well-posedness in Theorem 1.2 is understood in the usual quasilinear fashion, namely:

- Existence of solutions $(r,v) \in C([0,T],\mathbf {H}^{2k})$.
- Uniqueness of solutions in a larger class, see Theorem 1.1.
- Continous dependence of solutions on the initial data in the $\mathbf {H}^{2k}$ topology.

Furthermore, in our proof of uniqueness in Section 4 we establish something stronger, namely, that a suitable nonlinear distance between two solutions is propagated under the flow. This distance functional, in particular, tracks the distance between the boundaries of the moving domains associated with different solutions. Thus, our local well-posedness also includes:

- Weak Lipschitz dependence on the initial data relative to a suitable nonlinear functional introduced in Section 4.

An important threshold for our results corresponds to the uniform control parameters *A* and *B*. Of these *A* is at scaling, while *B* is one half of a derivative above scaling. Thus, by Lemma 2.5 of Section 2, we will have the bounds

$$\begin{aligned} A \lesssim \Vert (r,v)\Vert _{{\mathbf{H} }^{2k}}, \qquad k > k_0 = \frac{d+1}{2} +\frac{1}{2\kappa }, \end{aligned}$$

and

$$\begin{aligned} \qquad B \lesssim \Vert (r,v)\Vert _{{\mathbf{H} }^{2k}}, \qquad k > k_0+\frac{1}{2} = \frac{d+2}{2} +\frac{1}{2\kappa }. \end{aligned}$$

Next, we turn our attention to the continuation of solutions.

#### Theorem 1.3

For each integer $k\ge 0$ there exists an energy functional $E^{2k} = E^{2k}(r,v)$ with the following properties:

a) Coercivity: as long as *A* remains bounded, we have

$$\begin{aligned} E^{2k}(r,v) \approx \Vert (r,v)\Vert ^2_{\mathcal {H}^{2k}}. \end{aligned}$$

b) Energy estimates hold for solutions to (1.16), i.e.

$$\begin{aligned} \frac{d}{dt} E^{2k}(r,v) \lesssim _A B \Vert (r,v)\Vert ^2_{\mathcal {H}^{2k}}. \end{aligned}$$

By Gronwall’s inequality, Theorem 1.3 readily implies

1.28 $$\begin{aligned} \Vert (r,v)(t)\Vert ^2_{\mathcal {H}^{2k}} \lesssim e^{\int _0^t C(A) B(\tau ) \, d\tau } \Vert (\mathring{r},\mathring{v})\Vert _{\mathcal {H}^{2k}}^2, \end{aligned}$$

where *C*(*A*) is a constant depending on *A*. The energies $E^{2k}$ will be constructed explicitly only for integer *k*. Nevertheless, our analysis will show that (1.28) will also hold for any $k>0$. This will be done using a mechanism akin to a paradifferential expansion, without explicitly constructing energy functionals for non-integer *k*. As a consequence, we will obtain

#### Theorem 1.4

Let *k* be as in (1.27). Then, the $\mathbf {H}^{2k}$ solution given by Theorem 1.2 can be continued as long as *A* remains bounded and $B \in L^1_t(\Omega )$.

### Historical comments

The study of the relativistic Euler equations goes back to the early days of relativity theory, with the works of Einstein [7] and Schwarzschild [38]. The relativistic free-boundary Euler equations were introduced in the ’30s in the classical works of Tolman, Oppenheimer, and Volkoff [34, 39, 40], where they derived the now-called TOV equations.^8^ With the goal of modeling a star in the framework of relativity, Tolman, Oppenheimer, and Volkoff studied spherically symmetric static solutions to the Einstein-Euler system for a fluid body in vacuum and identified the vanishing of the pressure as the correct physical condition on the boundary. Observe that such a condition covers both the cases of a liquid, where $\varrho >0$ on the boundary, as well as a gas, which we study here, where $\varrho =0$ on the boundary. This distinction is related to the choice of equation of state.

Although the TOV equations have a long history and the study of relativistic stars is an active and important field of research (see, e.g., [37], Part III and [30], Part V), the mathematical theory of the relativistic free-boundary Euler equations lagged behind.

If we restrict ourselves to spherically-symmetric solutions, possibly also considering coupling to Einstein’s equations, a few precise and satisfactory mathematical statements can be obtained. Lindblom [20] proved that a static, asymptotically flat spacetime, that contains only a uniform-density perfect fluid confined to a spatially compact region ought to be spherically symmetric, thus generalizing to relativity a classical result of Carleman [4] and Lichtenstein [19] for Newtonian fluids. The proof of existence of spherically symmetric static solutions to the Einstein-Euler system consisting of a fluid region and possibly a vacuum region was obtained by Rendall and Schmidt [36]. Their solutions allow for the vanishing of the density along the interface of the fluid-vacuum region, although it is also possible that the fluid occupies the entire space and the density merely approaches zero at infinity. Makino [21] refined this result by providing a general criterion for the equation of state which ensures that the model has finite radius. Makino has also obtained solutions to the Einstein-Euler equations in spherical symmetry with a vacuum boundary and near equilibrium in [22, 23], where equilibrium here corresponds to the states given by the TOV equations. In [24], Makino extended these results to axisymmetric solutions that are slowly rotating, i.e., when the speed of light is sufficiently large or when the gravitational field is sufficiently weak (see also the follow-up works [25, 26] and the preceding work in [13]). Another result within symmetry class related to the existence of vacuum regions and relevant for the mathematical study of star evolution is Hadžić and Lin’s recent proof of the “turning point principle” for relativistic stars [11].

The discussion of the last paragraph was not intended to be an exhaustive account of the study of the relativistic free-boundary Euler equations under symmetry assumptions, and we refer the reader to the above references for further discussion. Rather, the goal was to highlight that a fair amount of results can be obtained in symmetry classes. This is essentially because some of the most challenging aspects of the problem are absent or significantly simplified when symmetry is assumed. This should be contrasted with what is currently known in the general case, which we now discuss.

Local existence and uniqueness of solutions the relativistic Euler equations in Minkowski background with a compactly supported density have been obtained by Makino and Ukai [27, 28] and LeFloch and Ukai [18]. These solutions, however, require some strong regularity of the fluid variables near the free boundary and, in particular, do not allow for the existence of physical vacuum states. Similarly, Rendall [35] established a local existence and uniqueness^9^ result for the Einstein-Euler system where the density is allowed to vanish. Nevertheless, as the author himself pointed out, the solutions obtained are not allowed to accelerate on the free boundary and, in particular, do not include the physical vacuum case. Rendall’s result has been improved by Brauer and Karp [2, 3], but still without allowing for a physical vacuum boundary. Oliynyk [31] was able to construct solutions that can accelerate on the boundary, but his result is valid only in one spatial dimension. A new approach to investigate the free-boundary Euler equations, based on a frame formalism, has been proposed by Friedrich in [8] (see also [9]) and further investigated by the first author in [6], but it has not led to a local well-posedness theory.

In the case of a *liquid*, i.e., where the fluid has a free-boundary where the pressure vanishes but the density remains strictly positive, a-priori estimates have been obtained by Ginsberg [10] and Oliynyk [32]. Local existence of solutions was recently established by Oliynyk [33] whereas Miao, Shahshahani, and Wu [29] proved local existence and uniqueness for the case when the fluid is in the so-called hard phase, i.e., when the speed of sound equals to one. See also [41], where the author, after providing a proof of local existence for the non-isentropic compressible free-boundary Euler equations in the case of a liquid, discusses ideas to adapt his proof for the relativistic case.

Finally, for the case treated in this paper, i.e., the relativistic Euler equations with a *physical vacuum* boundary, the only results we are aware of are the a-priori estimates by Hadžić, Shkoller, and Speck [12] and Jang, LeFloch, and Masmoudi [15]. In particular, no local existence and uniqueness (let along a complete local well-posedness theory as we present here) had been previously established.

### Outline of the paper

Our approach carefully considers the dual role of *r*, on the one hand, as a dynamical variable in the evolution and, on the other hand, as a defining function of the domain that, in particular, plays the role of a weight in our energies. An important aspect of our approach is to decouple these two roles. Such decoupling is what allows us to work entirely in Eulerian coordinates. When comparing different solutions (which in general will be defined in different domains), we can think of the role of *r* as a defining function as leading to a measure of the distance between the two domains (i.e., a distance between the two boundaries), whereas the role of *r* as a dynamical variable leads to a comparison in the common region defined by the intersection of the two domains. For instance, in our regularization procedure for the construction of regular solutions, the defining functions of the domains are regularized at a different scale than the main dynamical variables.

Although the relativistic and non-relativistic Euler equations, and their corresponding physical vacuum dynamics, are very different, some of our arguments here will closely follow those in the last two authors’ prior work [14], where results similar to those of Section 1.5 were established for the non-relativistic Euler equations in physical vacuum. Thus, when it is appropriate, we will provide a brief proof, or quote directly from [14]. This is particularly the case for Sects. 6 and 7.

The paper is organized as follows:

#### Function spaces, Sect. 2

This section presents the functional framework needed to study Eq. (1.16). These are spaces naturally associated with the degenerate wave operator

$$\begin{aligned} D_{t}^2 - r G^{ij} \partial _i \partial _j \end{aligned}$$

that is key to our analysis. Similar scales of spaces have been introduced in [14] treating the non-relativistic case and also in [16] where the non-relativistic problem had been considered in Lagrangian coordinates and in high regularity spaces.

Our function spaces $\mathcal {H}^{2k}$ are Sobolev-type spaces with weights *r*. Since the fluid domain is determined by $\Omega _t{:=} \left\{ r>0\right\}$, the state space $\mathbf {H}^{2k}$ is nonlinear, having a structure akin to an infinite dimensional manifold.

Interpolation plays two key roles in our work. Firstly, it allows us to define $\mathcal {H}^{2k}$ for non-integer *k* without requiring us to establish direct energy estimates with fractional derivatives. This is in particular important for our low regularity setting since the critical exponent (1.24) will in general not be an integer. Secondly, we interpolate between $\mathcal {H}^{2k}$ and the control norms *A* and *B*. For this we use some sharp interpolation inequalities presented in Section 2.3. These inequalities are proven in the last two authors’ prior work [14] and, to the best of our knowledge, have not appeared in the literature before. In fact, it is the use of these inequalities that allows us to work at low regularity, to obtain sharp energy estimates, and a continuation criterion at the level of scaling.

#### The linearized equation and the corresponding transition operators, Sect. 3

The linearized equation and its analysis form the foundation of our work, rather than direct nonlinear energy estimates. Besides allowing us to prove nonlinear energy estimates for single solutions, basing our analysis on the linearized equation will also allow us to get good quantitative estimates for the difference of two solutions. The latter is important for our uniqueness result and for the construction of rough solutions as limits of smooth solutions. We observe that there are no boundary conditions that need to be imposed on the linearized variables. This is related to the aforementioned decoupling of the roles of *r* and signals a good choice of functional framework.

Using the linearized equation we obtain transition operators $L_1$ and $L_2$ that act at the level of the linearized variables *s* and *w*. These transition operators are roughly the leading elliptic part of the wave equations for *s* and the divergence part of *w*. Note that since the wave evolution for the fluid degenerates on the boundary due to the vanishing of the sound speed, so do the transition operators $L_1$ and $L_2$. We refer to $L_1$ and $L_2$ as transition operators because they relate the spaces $\mathcal {H}^{2k+2}$ and $\mathcal {H}^{2k}$ in a coercive, invertible manner. Because of that, these operators play an important role in our regularization scheme used to construct high-regularity solutions.

#### Difference estimates and uniqueness, Sect. 4

In this section we construct a nonlinear functional that allows us to measure the distance between two solutions. We show that bounds for this functional are propagated by the flow, which in particular implies uniqueness. A fundamental difficulty is that, since we are working in Eulerian coordinates, different solutions are defined in different domains. This difficulty is reflected in the nonlinear character of our functional, which could be thought of as measuring the distance between the boundaries of two different solutions. The low regularity at which we aim to establish uniqueness leads to some technical complications that are dealt with by a careful analysis of the problem.

#### Energy estimates and coercivity, Sect. 5

The energies that we use contain two components, a wave component and a transport component, in accordance with the wave-transport character of the system. The energy is constructed after identifying Alinhac-type “good variables” that can be traced back to the structure of the linearized problem. This connection with the linearized problem is also key to establish the coercivity of the energy in that it relies on the transition operators $L_1$ and $L_2$ mentioned above.

#### Existence of regular solutions, Sect. 6

This section establishes the existence of regular solutions. It heavily relies on the last two authors’ prior work [14], to which the reader is referred for several technical points.

Our construction is based essentially on an Euler scheme to produce good approximating solutions. Nevertheless, a direct implementation of Euler’s method loses derivatives. We overcome this by preceding each iteration with a regularization at an appropriate scale and a separate transport step. The main difficulty is to control the growth of the energies at each step.

#### Rough solutions as limits of regular solutions, Sect. 7

In this section we construct rough solutions as limits of smooth solutions, in particular establishing the existence part of Theorem 1.2. We construct a family of dyadic regularizations of the data, and control the corresponding solutions in higher ${\mathcal H }^{2k}$ norms with our energy estimates, and the difference of solutions in $\mathcal {H}$ with our nonlinear stability bounds. The latter allow us to establish the convergence of the smooth solutions to the desired rough solution in weaker topologies. Convergence in ${\mathbf{H} }^{2k}$ is obtained with more accurate control using frequency envelopes. A similar argument then also gives continuous dependence on the data.

### Notation for $v,\omega$ and the use of Latin indices

In view of Eq. (1.16) and the corresponding vorticity evolution (1.22), we have now written the dynamics solely in terms of *r* and the spatial components of *v*, i.e., $v^i$. We henceforth consider *v* as a *d*-dimensional vector field, so that whenever referring to *v* we always mean $(v^1,\dots ,v^d)$. $v^0$ is always understood as a shorthand for the RHS of (1.17). Similarly, by $\omega$ will stand for $\omega _{ij}$.

Recalling that indices are raised and lowered with the Minkowski metric and that $m_{0i}= 0 = m^{0i}$, $m_{ij} = \updelta _{ij}$, we see that tensors containing only Latin indices have indices equivalently raised and lowered with the Euclidean metric.

## Function spaces

Here we define the function spaces that will play a role in our analysis. They are weighted spaces with weights given by the sound speed squared *r* which, in view of (1.25), is comparable to the distance to the boundary. More precisely, since a solution to (1.16) is not a-priori given, in the definitions below we take *r* to be a fixed non-degenerate defining function for the domain $\Omega _t$, i.e., proportional to the distance to the boundary $\Gamma _t$. In turn, the boundary $\Gamma _t$ is assumed to be Lipschitz.

We denote the $L^2$-weighted spaces with weights *h* by $L^2(h)$ and we equip them with the norm

$$\begin{aligned} \Vert f\Vert _{L^2(h)} {:=} \int _{\Omega _t} h |f|^2 \, dx. \end{aligned}$$

With these notations the base $L^2$ space of pairs of functions in $\Omega$ for our system, denoted by ${\mathcal H }$, is defined as

$$\begin{aligned} {\mathcal H }= L^2(r^{\frac{1-\kappa }{\kappa }}) \times L^2(r^{\frac{1}{k}}). \end{aligned}$$

This space depends only on the choice of *r*. However, we will often use an equivalent norm that also depends on *v*, which corresponds to the energy space for the linearized problem and will also be important in the construction of our energies:

2.1 $$\begin{aligned} \Vert (s,w)\Vert ^2_{\mathcal {H}} = \int _{\Omega _t} r^{\frac{1-\kappa }{\kappa }} ( s^2 + a_2^{-1} r G^{ij} w_i w_j )\, dx. \end{aligned}$$

This uses $G$ to measure the pointwise norm of the one form *w*. The $\mathcal {H}$ norm is equivalent to the $\mathcal {H}^0$ norm (see the definition of $\mathcal {H}^{2k}$ below) since $G$ is equivalent to the the Euclidean inner product with constants depending on the $L^\infty$ norm of (*r*, *v*).

We continue with higher Sobolev norms. We define $H^{j,\sigma }$, where $j\ge 0$ is an integer and $\sigma > -\frac{1}{2}$, to be the space of all distributions in $\Omega _t$ whose norm

$$\begin{aligned} \Vert f \Vert ^2_{H^{j,\sigma }} {:=} \sum _{ \vert \alpha \vert \le j} \Vert r^\sigma \partial ^{\alpha }f \Vert _{L^2}^2 \end{aligned}$$

is finite. Using interpolation, we extend this definition, thus defining $H^{s,\sigma }$ for all real $s \ge 0$.

To measure higher regularity we will also need higher Sobolev spaces where the weights depend on the number of derivatives. More precisely, we define $\mathcal {H}^{2k}$ as the space of pairs of functions (*s*, *w*) defined inside $\Omega _t$, and for which the norm below is finite :

$$\begin{aligned} \Vert (s,w)\Vert ^2_{\mathcal {H}^{2k}} := \sum _{\vert \alpha \vert =0}^{2k} \sum _{\begin{array}{c} a=0 \\ |\alpha |- a\le k \end{array}}^{k} \Vert r^{\frac{1-\kappa }{2\kappa } + a} \partial ^\alpha s\Vert ^2_{L^2} + \sum _{\vert \alpha \vert =0}^{2k} \sum _{\begin{array}{c} a=0 \\ |\alpha |- a\le k \end{array}}^{k} \Vert r^{\frac{1-\kappa }{2\kappa }+\frac{1}{2} + a} \partial ^\alpha w \Vert ^2_{L^2}. \end{aligned}$$

We extend the definition of $\mathcal {H}^{2k}$ to non-integer *k* using interpolation. An explicit characterization of $\mathcal {H}^{2k}$ for non-integer *k*, based on interpolation, was given in the last two authors’ prior work [14]. Using the embedding theorems given below, we can show that the $\mathcal {H}^{2k}$ norm is equivalent to the $H^{2k,\frac{1-\kappa }{2\kappa }+k} \times H^{2k,\frac{1}{2\kappa }+k}$ norm.

### The state space ${\mathbf{H} }^{2k}$.

As already mentioned in the introduction, the state space ${\mathbf{H} }^{2k}$ is defined for $k > k_0$ (i.e. above scaling) as the set of pairs of functions (*r*, *v*) defined in a domain $\Omega _t$ in ${\mathbb R}^d$ with boundary $\Gamma _t$ with the following properties:

- Boundary regularity: $\Gamma _t$ is a Lipschitz surface.
- Nondegeneracy: *r* is a Lipschitz function in $\bar{\Omega }_t$, positive inside $\Omega _t$ and vanishing simply on the boundary $\Gamma _t$.
- Regularity: The functions (*r*, *v*) belong to ${\mathcal H }^{2k}$.

Since the domain $\Omega _t$ itself depends on the function *r*, one cannot think of ${\mathbf{H} }^{2k}$ as a linear space, but rather as an infinite dimensional manifold. However, describing a manifold structure for ${\mathbf{H} }^{2k}$ is beyond the purposes of our present paper, particularly since the trajectories associated with our flow are merely expected to be continuous with values in ${\mathbf{H} }^{2k}$. For this reason, here we will limit ourselves to defining a topology on ${\mathbf{H} }^{2k}$.

#### Definition 2.1

A sequence $(r_n,v_n)$ converges to (*r*, *v*) in ${\mathbf{H} }^{2k}$ if the following conditions are satisfied:

i) Uniform nondegeneracy, $$|\nabla r_n| \ge c > 0$$.
ii) Domain convergence, $\Vert r_n - r\Vert _{Lip} \rightarrow 0$. Here, we consider the functions $r_n$ and and *r* as extended to zero outside their domains, giving rise to Liptschitz functions in ${\mathbb R}^d$.
iii) Norm convergence: for each $\epsilon > 0$ there exist a smooth function $(\tilde{r},\tilde{v})$ in a neighbourhood of $\Omega$ so that
  $$\begin{aligned} \Vert (r,v)- (\tilde{r},\tilde{v})\Vert _{{\mathcal H }^{2k}(\Omega )} \le \epsilon , \qquad \limsup _{n \rightarrow \infty } \Vert (r_n, v_n)- (\tilde{r},\tilde{v})\Vert _{{\mathcal H }^{2k}(\Omega _n)} \le \epsilon . \end{aligned}$$

The last condition in particular provides both a uniform bound for the sequence $(r_n,v_n)$ in ${\mathcal H }^{2k}(\Omega _n)$ as well as an equicontinuity type property, insuring that a nontrivial portion of their ${\mathcal H }^{2k}$ norms cannot concentrate on thinner layers near the boundary. This is akin to the the conditions in the Kolmogorov-Riesz theorem for compact sets in $L^p$ spaces.

This definition will enable us to achieve two key properties of our flow:

- Continuity of solutions (*r*, *v*) as functions of *t* with values in ${\mathbf{H} }^{2k}$.
- Continuous dependence of solutions $(r,v) \in C ({\mathbf{H} }^{2k})$ as functions of the initial data $(\mathring{r},\mathring{v}) \in {\mathbf{H} }^{2k}$.

### Regularization and good kernels

In what follows we outline the main steps developed in Section 2 of [14], and in which, for a given state (*r*, *v*) in ${\mathbf{H} }^{2k}$, we construct regularized states, denoted by $(r^h, v^h)$, to our free boundary evolution, associated to a dyadic frequency scale $2^h$, $h \ge 0$. This relies on having good regularization operators associated to each dyadic frequency scale $2^h$, $h \ge 0$. We denote these regularization operators by $\Psi ^h$, with kernels $K^h$. These are the same as in [14], and their exact definition can be found in there as well. A brief description on how one should envision these regularization operators is in order.

It is convenient to think of the domain $\Omega _t$ as partitioned in dyadic boundary layers, denoting by $\Omega ^{[j]}$ the layer at distance $2^{-2j}$ away from the boundary. Within each boundary layer we need to understand which is the correct spatial regularization scale. The principal part of the second order elliptic differential operator associated to our system is the starting point. Given a dyadic frequency scale *h*, our regularizations will need to select frequencies $\xi$ with the property that $r \xi ^2 \lesssim 2^{2h}$, which would require kernels on the dual scale

$$\begin{aligned} \delta x \approx r^{\frac{1}{2}} 2^{-h}. \end{aligned}$$

However, if we are too close to the boundary, i.e. $r \ll 2^{-2h}$, then we run into trouble with the uncertainty principle, as we would have $\delta x \gg r$. To remedy this issue we select the spatial scale $r \lesssim 2^{-2h}$ and the associated frequency scale $2^{2h}$ as cutoffs in this analysis. Then the way the regularization works is as follows: (i) for $j < h$, the regularizations $(r^h,v^h)$ in $\Omega ^{[j]}$ are determined by (*r*, *v*) also in $\Omega ^{[j]}$, and (ii) for $j=h$, the values of (*r*, *v*) in $\Omega ^{[h]}$ determine $(r^h, v^h)$ in a full neighborhood $\tilde{\Omega }^{[>h]}$ of $\Gamma$, of size $2^{-2h}$. The regularized state is obtained by restricting the full regularization to the domain $\Omega _h {:=}\left\{ r^h>0\right\}$.

For completeness we state the result in [14], and refer the reader there for the proof:

#### Proposition 2.2

Assume that $k > k_0$. Then given a state $(r,v) \in {\mathbf{H} }^{2k}$, there exists a family of regularizations $(r^{h},v^h) \in {\mathbf{H} }^{2k}$, so that the following properties hold for a slowly varying frequency envelope $c_h \in \ell ^2$ which satisfies

2.2 $$\begin{aligned} \Vert c_h\Vert _{\ell ^2} \lesssim _A \Vert (r,v) \Vert _{{\mathbf{H} }^{2k}} . \end{aligned}$$

i) Good approximation,
  2.3 $$\begin{aligned} (r^{h},v^h) \rightarrow (r,v) \quad \text { in } C^1 \times C^\frac{1}{2} \quad \text { as } h \rightarrow \infty , \end{aligned}$$
  and
  2.4 $$\begin{aligned} \Vert r^{h} - r\Vert _{L^\infty (\Omega )} \lesssim 2^{-2(k-k_0+1) h }. \end{aligned}$$
ii) Uniform bound,
  2.5 $$\begin{aligned} \Vert (r^h,v^h) \Vert _{{\mathbf{H} }^{2k}} \lesssim _{A} \Vert (r,v) \Vert _{{\mathbf{H} }^{2k}} . \end{aligned}$$
iii) Higher regularity
  2.6 $$\begin{aligned} \Vert (r^h,v^h) \Vert _{{\mathbf{H} }^{2k+2j}_h} \lesssim 2^{2hj} c_h, \qquad j > 0. \end{aligned}$$
iv) Low frequency difference bound:
  2.7 $$\begin{aligned} \Vert (r^{h+1},v^{h+1}) - (r^h,v^h) \Vert _{{\mathcal H }_{\tilde{r}}} \lesssim 2^{-2hk} c_h, \end{aligned}$$
  for any defining function $\tilde{r}$ with the property $|\tilde{r} - r| \ll 2^{-2h}.$

### Embedding and interpolation theorems

In this section we state some embedding and interpolation results that will be used throughout.They have been proved in the last two authors’ prior paper [14], to which the reader is referred to for the proofs.

#### Lemma 2.3

Assume that $s_1 > s_2 \ge 0$ and $\sigma _1> \sigma _2 > -\frac{1}{2}$ with $s_1-s_2 = \sigma _1-\sigma _2$. Then we have

$$\begin{aligned} H^{s_1,\sigma _1} \subset H^{s_2,\sigma _2}. \end{aligned}$$

As a corollary of the above lemma we have embeddings into standard Sobolev spaces.

#### Lemma 2.4

Assume that $\sigma > 0$ and $\sigma \le j$. Then we have

$$\begin{aligned} H^{j,\sigma } \subset H^{j-\sigma }. \end{aligned}$$

In particular, by standard Sobolev embeddings, we also have Morrey type embeddings into $C^s$ spaces:

#### Lemma 2.5

We have

$$\begin{aligned} H^{j,\sigma } \subset C^{s}, \qquad 0 \le s \le j- \sigma - \frac{d}{2}, \end{aligned}$$

where the equality can hold only if *s* is not an integer.

Next, we state the interpolation bounds.

#### Proposition 2.6

Let $\sigma _0, \sigma _m \in \mathbb {R}$ and $1\le p_0,p_m\le \infty$. Define

$$\begin{aligned} \theta _j = \frac{j}{m}, \qquad \frac{1}{p_j} = \frac{1-\theta _j}{p_0}+\frac{\theta _j}{p_m}, \qquad \sigma _j = \sigma _0(1-\theta _j) + \sigma _m\theta _j, \end{aligned}$$

and assume that

$$\begin{aligned} m - \sigma _m - d\left( \frac{1}{p_m}-\frac{1}{p_0} \right)> -\sigma _0, \qquad \sigma _j >-\frac{1}{p_j}. \end{aligned}$$

Then for $0< j < m$ we have

$$\begin{aligned} \Vert r^{\sigma _j } \partial ^j f \Vert _{L^{p_j}} \lesssim \Vert r^{\sigma _0} f \Vert _{L^{p_0}}^{1-\theta _j} \Vert r^{\sigma _m}\partial ^m f \Vert _{L^{p_m}}^{\theta _j} . \end{aligned}$$

#### Remark 2.7

One particular case of the above proposition which will be used later is when $p_0=p_1=p_2=2$, with the corresponding relation in between the exponents of the $r^{\sigma _j}$ weights.

As the objective here is to interpolate between the $L^2$ type $H^{m,\sigma }$ norm and $L^\infty$ bounds, we will need the following straightforward consequence of Proposition 2.6:

#### Proposition 2.8

Let $$\sigma _m > -\frac{1}{2}$$ and

$$\begin{aligned} m - \sigma _m - \frac{d}{2} > 0. \end{aligned}$$

Define

$$\begin{aligned} \theta _j = \frac{j}{m}, \qquad \frac{1}{p_j} = \frac{\theta _j}{2}, \qquad \sigma _j = \sigma _m \theta _j. \end{aligned}$$

Then for $0< j < m$ we have

$$\begin{aligned} \Vert r^{\sigma _j } \partial ^j f \Vert _{L^{p_j}} \lesssim \Vert f \Vert _{L^\infty }^{1-\theta _j} \Vert r^{\sigma _m}\partial ^{m}f \Vert _{L^{2}}^{\theta _j} . \end{aligned}$$

We will also need the following two variations of Proposition 2.8:

#### Proposition 2.9

Let $$\sigma _m >-\frac{1}{2}$$ and

$$\begin{aligned} m-\frac{1}{2}-\sigma _m -\frac{d}{2}>0. \end{aligned}$$

Define

$$\begin{aligned} \sigma _j=\sigma _m\theta _j,\quad \theta _j=\frac{2j-1}{2m-1}, \quad \frac{1}{p_j}=\frac{\theta _j}{2}. \end{aligned}$$

Then for $0<j<m$ we have

$$\begin{aligned} \Vert r^{\sigma _j}\partial ^j f\Vert _{L^{p_j}}\lesssim \Vert f\Vert ^{1-\theta _j}_{\dot{C}^{\frac{1}{2}}} \Vert r^{\sigma _m}\partial ^m f \Vert ^{\theta _j}_{L^{2}}. \end{aligned}$$

#### Proposition 2.10

Let $$\sigma _m >\frac{m-2}{2}$$ and

$$\begin{aligned} m-\frac{1}{2}-\sigma _m -\frac{d}{2}>0. \end{aligned}$$

Define

$$\begin{aligned} \sigma _j=\sigma _m\theta _j-\frac{1}{2}(1-\theta _j),\quad \theta _j=\frac{j}{m}, \quad \frac{1}{p_j}=\frac{\theta _j}{2}. \end{aligned}$$

Then for $0<j<m$ we have

$$\begin{aligned} \Vert r^{\sigma _j}\partial ^j f\Vert _{L^{p_j}}\lesssim \Vert f\Vert ^{1-\theta _j}_{\tilde{C}^\frac{1}{2}} \Vert r^{\sigma _m}\partial ^m f \Vert ^{\theta _j}_{L^{2}}. \end{aligned}$$

## The linearized equation

Consider a one-parameter family of solutions $(r_\tau , v_\tau )$ for the main system (1.16) such that $\left. (r_\tau ,v_\tau )\right| _{\tau =0} = (r,v)$, Then formally the functions $\left. \frac{d}{d \tau } (r_\tau ,v_\tau )\right| _{\tau =0} = (s,w)$, defined in the moving domain $\Omega _t$, will solve the corresponding linearized equation. Precisely, a direct computation shows that, for (*s*, *w*) in $\Omega _t$, the linearized equation can be written in the form
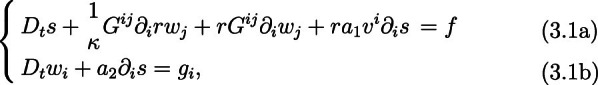


where *f* and $g_i$ represent perturbative terms of the form

$$\begin{aligned} f = V_1 s + r W_1 w, \qquad g = V_2 s + W_2 w \end{aligned}$$

with potentials $V_{1,2}$ and $W_{1,2}$ which are linear in $\partial (r,v)$, with coefficients which are smooth functions of *r* and *v*.

Importantly, we remark that for the above system we do not obtain or require any boundary conditions on the free boundary $\Gamma _t$. This is related to the fact that our one parameter family of solutions are not required to have the same domain, as it would be the case if one were working in Lagrangian coordinates.

For completeness, we also provide the explicit expressions for the potentials $V_{1,2}$ and $W_{1,2}$, though this will not play any role in the sequel. We have

$$\begin{aligned} \begin{aligned} V_{1}&= \ \frac{v^j}{(v^0)^3}\langle r \rangle ^{1+\frac{2}{\kappa }} \partial _j r - G^{ij} \partial _i v_j - r \frac{\partial G^{ij}}{\partial r} \partial _i v_j ,\\ W_{2}^{l}&= \ - \frac{\partial G^{ij}}{\partial v^l} \partial _i v_j - r a_3 G^{il} \partial _i r ,\\ V_{2,i}&= -\frac{v^j}{(v^0)^3}\langle r \rangle ^{1+\frac{2}{\kappa }} \partial _j v_i + \frac{\partial a_2}{\partial r} \partial _i r ,\\ (W_{2})_i^l&= \ - \frac{a_0}{\kappa \langle r \rangle } G^{jl} \partial _j v_i + \frac{\partial a_2}{\partial v^l} \partial _i r , \end{aligned} \end{aligned}$$

where $a_3$ is a smooth function of (*r*, *v*), given by

3.2 $$\begin{aligned} \frac{a_0}{\kappa \langle r \rangle } - \frac{1}{\kappa } = r a_3,\qquad a_3 = - \frac{1}{\langle r \rangle } \left( \frac{1}{2} + \frac{|v|^2}{(v^0)^2} \right) . \end{aligned}$$

As for the other coefficients, the particular form of $a_3$ is not relevant, but we wrote it here for completeness.

### Energy estimates and well-posedness

We now consider the well-posedness of the linearized problem (3.1) in the time dependent space ${\mathcal H }$. For the purpose of this analysis, we will view ${\mathcal H }$ as a Hilbert space whose squared norm plays the role of the energy functional for the linearized equation,

3.3 $$\begin{aligned} E_{lin}(s,w) {:=} \Vert (s,w)\Vert ^2_{\mathcal {H}} = \int _{\Omega _t} r^{\frac{1-\kappa }{\kappa }} ( s^2 + a_2^{-1} r G^{ij} w_i w_j )\, dx. \end{aligned}$$

We will use this space for both the linearized equation and its adjoint. Our main result here is as follows:

#### Proposition 3.1

Let (*r*, *v*) be a solution to (1.16). Assume that both *r* and *v* are Lipschitz continuous and that *r* vanishes simply on the free boundary. Then, the linearized Eq. (3.1) are well-posed in $\mathcal {H}$, and the following estimate holds for solutions (*s*, *w*) to (3.1):

3.4 $$\begin{aligned} \left| \frac{d}{dt} \Vert (s,w)\Vert ^2_{{\mathcal {H}}} \right| \lesssim B \Vert (s,w)\Vert ^2_{{\mathcal {H}}}. \end{aligned}$$

#### Proof

We first remark that (*f*, *g*) are indeed perturbative terms, as they satisfy the estimate

3.5 $$\begin{aligned} \Vert (f, g)\Vert _{{\mathcal {H}} } \lesssim B \Vert (s, w)\Vert _{{\mathcal {H}}}. \end{aligned}$$

This in turn follows from a trivial pointwise bound on the corresponding potentials,

$$\begin{aligned} \Vert V_{1,2} \Vert _{L^\infty } + \Vert W_{1,2}\Vert _{L^\infty } \lesssim B. \end{aligned}$$

We multiply (3.1a) by $r^{\frac{1-\kappa }{\kappa }}s$ and contract (3.1b) with $a_2^{-1} r^{\frac{1}{\kappa }}G^{ij} w_j$ to find

$$\begin{aligned}&\frac{1}{2} r^\frac{1-\kappa }{\kappa } D_{t}s^2 + \frac{1}{\kappa } r^\frac{1-\kappa }{\kappa } {G}^{ij} \partial _i r w_j s + r^\frac{1}{\kappa } {G}^{ij} \partial _i w_j s +\frac{1}{2} r^\frac{1}{\kappa } a_1 v^i \partial _i s^2 = f r^\frac{1-\kappa }{\kappa } s,\\&\frac{1}{2}a_2^{-1} r^\frac{1}{\kappa } {G}^{ij} D_{t}(w_i w_j) + r^\frac{1}{\kappa } G^{ij}w_j \partial _i s = a_2^{-1} r^\frac{1}{\kappa } {G}^{ij} g_i w_j. \end{aligned}$$

Next, we add the two equations above, noting that the second and third terms on the LHS of the first equation combine with the second term on the LHS of the second equation to produce

$$\begin{aligned}&\frac{1}{\kappa } r^\frac{1-\kappa }{\kappa } {G}^{ij} \partial _i r w_j s + r^\frac{1}{\kappa } {G}^{ij} \partial _i w_j s + r^\frac{1}{\kappa } G^{ij} w_j \partial _i s \\&\quad = \partial _i(r^\frac{1}{\kappa } ){G}^{ij} w_j s + r^\frac{1}{\kappa } {G}^{ij} \partial _i w_j s + r^\frac{1}{\kappa } G^{ij} w_j \partial _i s\\&\quad = {G}^{ij} \partial _i (r^\frac{1}{\kappa } w_j s) \end{aligned}$$

This yields

$$\begin{aligned}&\frac{1}{2} r^\frac{1-\kappa }{\kappa } D_{t}s^2 +\frac{1}{2}a_2^{-1} r^\frac{1}{\kappa } {G}^{ij} D_{t}(w_i w_j) +\frac{1}{2} r^\frac{1}{\kappa } a_1 v^i \partial _i s^2 + {G}^{ij} \partial _i ( r^\frac{1}{\kappa } w_j s) \\&\quad = f r^\frac{1-\kappa }{\kappa } s + a_2^{-1} r^\frac{1}{\kappa } {G}^{ij} g_i w_j. \end{aligned}$$

We now integrate the above identity over $\Omega _t$, using the formula (1.8) to produce a time derivative of the energy. For this, we need to write the terms on the left as perfect derivatives or material derivatives. When we do so the zero order coefficients do not cause any harm. We only need to be careful with the terms where a derivative falls on $r^\frac{1-\kappa }{\kappa }$ because this could potentially produce a term with the wrong weight (i.e., one less power of *r*). However, this does not occur because we can solve for $D_{t}r$ in (1.16a):

$$\begin{aligned} D_{t}r^\frac{1-\kappa }{\kappa } = \frac{1-\kappa }{\kappa } r^{\frac{1}{\kappa } -2} D_{t}r = r^\frac{1-\kappa }{\kappa } O( \partial (r,v) ). \end{aligned}$$

Using the above observations, we obtain

$$\begin{aligned} {\begin{matrix} \left| \frac{d}{dt} \Vert (s,w)\Vert ^2_{\mathcal {H}} \right| \lesssim B \Vert (s,w)\Vert ^2_{\mathcal {H}} + \Vert (s,w)\Vert _{\mathcal {H}} \Vert (f,g)\Vert _{\mathcal {H}} \lesssim B \Vert (s,w)\Vert ^2_{\mathcal {H}}. \end{matrix}} \end{aligned}$$

We now compute the adjoint equation to (3.1) with respect to the duality relation defined by the ${\mathcal H }$ inner product determined by the norm (3.3). The terms *f* and *g* on the RHS of (3.1) are linear expressions in *s* and *rw* and and in *s* and *w*, respectively, with $\partial (r,v)$ coefficients. Thus, the source terms in the adjoint equation have the same structure as the original equation. Let us write the LHS of (3.1) as

$$\begin{aligned} D_{t}\begin{pmatrix} s\\ w \end{pmatrix} + \mathsf {A}^i \partial _i \begin{pmatrix} s\\ w \end{pmatrix} + \mathsf {B} \begin{pmatrix} s\\ w \end{pmatrix}, \end{aligned}$$

where

$$\begin{aligned} \mathsf {A}^i = \begin{bmatrix} a_1 r v^i &{} r G^{ij} \\ a_2 \updelta ^{il} &{} 0_{3\times 3} \end{bmatrix} \end{aligned}$$

and

$$\begin{aligned} \mathsf {B} = \begin{bmatrix} 0_{1\times 1} &{} \frac{1}{\kappa } G^{ij}\partial _i r \\ 0_{3\times 1} &{} 0_{3\times 3} \end{bmatrix}. \end{aligned}$$

With respect to the $\mathcal {H}$ inner product, the adjoint term corresponding to $\mathsf {A}^i \partial _i$ is

$$\begin{aligned} \tilde{\mathsf {A}}^i \partial _i = - \begin{bmatrix} a_1 r v^i &{} r G^{ij} \\ a_2 \updelta ^{il} &{} 0_{3\times 3} \end{bmatrix}\partial _i - \begin{bmatrix} 0 &{} \frac{1}{\kappa }G^{ij}\partial _i r \\ \frac{1}{\kappa } r^{-1} a_2 \partial _l r &{} 0_{3\times 3} \end{bmatrix} \end{aligned}$$

modulo terms that are linear expressions in $\tilde{s}$ and $r \tilde{w}$ and in $\tilde{s}$ and $\tilde{w}$ (with $\partial (r,v)$ coefficients) in the first and second components, respectively, where $\tilde{s}$ and $\tilde{w}$ are elements of the dual. Similarly, the adjoint term corresponding to $\mathsf {B}$ is

$$\begin{aligned} \tilde{\mathsf {B}} = \begin{bmatrix} 0_{1 \times 1} &{} 0_{1\times 3} \\ \frac{1}{\kappa } r^{-1} a_2 \partial _l r &{} 0_{3 \times 3} \end{bmatrix}. \end{aligned}$$

Combining these expressions, we see that the bad term on the lower left corner of the second matrix in $\tilde{\mathsf {A}}^i \partial _i$ cancels with the corresponding terms in $\tilde{B}$. Therefore, the adjoint problem is the same as the original one, modulo perturbative terms, and it therefore admits an energy estimate similar to the energy estimate (3.4) we have for the linearized Eq. (3.1).

In a standard fashion, the forward energy estimate for the linearized equation and the backward in time energy estimate for the adjoint linearized equation yield uniqueness, respectively existence of solutions for the linearized equation, as needed. This guarantees the well-posedness of the linearized problem.

### Second order transition operators

An alternative approach the linearized equations is to rewrite the linearized Eq. (3.1) as a second order system which captures the wave-like part of the fluid associated with the propagation of sound. Applying $D_{t}$ to (3.1a) and using (3.1b) and vice-versa, and ignoring perturbative terms, we find

3.6a $$\begin{aligned}&D_{t}^2 s \approx \hat{L}_1 s, \end{aligned}$$

3.6b $$\begin{aligned}&D_{t}^2 w_i \approx (\hat{L}_2 w)_i, \end{aligned}$$

where

3.7a $$\begin{aligned}&\hat{L}_1 s {:=} r \partial _i \left( a_2 G^{ij} \partial _j s\right) + \frac{a_2}{\kappa } G^{ij} \partial _i r \partial _j s, \end{aligned}$$

3.7b $$\begin{aligned}&(\hat{L}_2 w)_i {:=} a_2 \left( \partial _i (r G^{ml} \partial _m w_l) + \frac{1}{\kappa } G^{ml} \partial _m r\partial _i w_l \right) . \end{aligned}$$

Equations (3.6a) and (3.6b) are akin to wave equations in that the operators $\hat{L}_1$ and $\hat{L}_2$ satisfy elliptic estimates as proved in Section 5.2. More precisely, the operator $\hat{L}_2$ is associated with the divergence part of *w*, and it satisfies elliptic estimates once it is combined with a matching curl operator $\hat{L}_3$.

Even though in this paper we do not use the operators $\hat{L}_1$ and $\hat{L}_2$ directly in connection to the corresponding wave equation, they nevertheless play an important role at two points in our proof. Because slightly different properties of $\hat{L}_1$ and $\hat{L}_2$ are needed at these two points, we will take advantage of the fact that only their principal part is uniquely determined in order to make slightly different choices for $\hat{L}_1$ and $\hat{L}_2$. Precisely, these operators will be needed as follows:

I. In the proof of our energy estimates in Section 5.2, in order to establish the coercivity of our energy functionals. There we will need the coercivity of $\hat{L}_1$ and $\hat{L}_2 + \hat{L}_3$, but we also want their coefficients to involve only $r,\nabla r$ and undifferentiated *v*.
II. In the constructive proof of existence of regular solutions, in our paradifferential style regularization procedure. There we use functional calculus for both $\hat{L}_1$ and $\hat{L}_2 + \hat{L}_3$, so we need them to be both coercive and self-adjoint, but we no longer need to impose the previous restrictions on the coefficients.

The two sets of requirements are not exactly compatible, which is why two choices are needed.^10^

We begin with the case (I), where we modify $\hat{L}_1$ and $\hat{L}_2$ as follows:

3.8a $$\begin{aligned}&\tilde{L}_1 s {:=} G^{ij} a_2 \left( r\partial _i \partial _j s + \frac{1}{\kappa } \partial _i r \partial _j s \right) , \end{aligned}$$

3.8b $$\begin{aligned}&(\tilde{L}_2 w)_i {:=} a_2 G^{ml} \left( \partial _i (r \partial _m w_l) + \frac{1}{\kappa } \partial _m r\partial _i w_l \right) . \end{aligned}$$

To $\tilde{L}_2$ we associate an operator $\tilde{L}_3$ of the form

3.9 $$\begin{aligned} ({\tilde{L}}_3 w)^i {:=} r^{-\frac{1}{\kappa }} a_2 G^{ij} \partial ^l [ r^{1+\frac{1}{\kappa }} (\partial _l w_j - \partial _j w_l) ]. \end{aligned}$$

For case (II), we keep the first of the operators, setting $L_1 = \hat{L}_1$ but make some lower order changes to $\hat{L}_2$ and $\hat{L}_3$ as follows:

3.10 $$\begin{aligned}&({L}_2 w)_i {:=} \partial _i\left( a_2^2 (r \partial _m + \frac{1}{\kappa } \partial _m r)(a_2^{-1} G^{ml} w_l)\right) . \end{aligned}$$

3.11 $$\begin{aligned}&(L_3 w)_i {:=} r^{-\frac{1}{\kappa }} a_2 F_{ij} \partial _l [ G^{lm} G^{jp} r^{1+\frac{1}{\kappa }} (\partial _m w_p - \partial _p w_m) ], \end{aligned}$$

where $F$ is the inverse of the matrix ${G}$, i.e., $F= {G}^{-1}$.

It is not difficult to show that $L_1$ is a self-adjoint operator in $L^2(r^\frac{1-\kappa }{\kappa })$ with respect to the inner product defined by the first component of the ${\mathcal H }$ norm in (2.1), and

$$\begin{aligned} \mathcal {D}(L_1) = \left\{ f \in L^2(r^{\frac{1-\kappa }{\kappa }}) \, | \, L_1 f \in L^2(r^{\frac{1-\kappa }{\kappa }}) \text { in the sense of distributions} \right\} . \end{aligned}$$

Similarly, both $L_2$ and $L_3$ are self-adjoint operators in $L^2(r^\frac{1}{\kappa })$ with respect to the inner product defined by the second component of the ${\mathcal H }$ norm in (2.1) and

$$\begin{aligned} \mathcal {D}(L_2) = \left\{ f \in L^2( r^{\frac{1}{\kappa }} ) \, | \, L_2 f \in L^2( r^{\frac{1}{\kappa }} ) \text { in the sense of distributions} \right\} . \end{aligned}$$

and similarly for $L_3$. We further note that $L_2L_3= L_3L_2=0$ and that the output of $L_2$ is a gradient, whereas $L_3 w$ depends only on the curl of *w*.

As seen above, it is the operator $\hat{L}_2$ that naturally come out of the equations of motion rather than $L_2$ (recall that $L_1 = \hat{L}_1$). Thus, we need to compare these operators; we also compare $L_1$ and $\tilde{L}_1$ for later reference. We have

3.12 $$\begin{aligned} {} \left\{ \begin{aligned}&(L_2 w)_i = (\hat{L}_2 w)_i + \partial _i a_2 r \partial _m ({G}^{ml} w_l) + \partial _i (r a_2^3 \partial _m a_2^{-1} {G}^{ml} w_l) +\frac{a_2}{\kappa } \partial _i ({G}^{ml} \partial _m r) w_l\\&L_1 s = \tilde{L}_1 s + r \partial _i(a_2 {G}^{ij}) \partial _j s. \end{aligned} \right. \end{aligned}$$

We will establish coercive estimates for $L_1$, $L_2$, and $L_3$ (see Sects. 5 and 6), from which follows that the above domains can be characterized as

$$\begin{aligned} \mathcal {D}(L_1) = H^{2,\frac{1+\kappa }{2\kappa }},\qquad \mathcal {D}(L_2+L_3) = H^{2,\frac{1+3\kappa }{2\kappa }}. \end{aligned}$$

## The uniqueness theorem

In this Section we establish Theorem 1.1. It will be a direct consequence of the more general Theorem 4.2 below, which establishes that a suitable functional that measures the difference between two solutions is propagated by the flow.

We consider two solutions $(r_1,v_1)$ and $(r_2,v_2)$ defined in the respective domains $\Omega ^1_t$ and $\Omega ^2_t$. Put $\Omega _t{:=} \Omega _t^1 \cap \Omega _t^2$, $\Gamma _t{:=} \partial \Omega _t$. If the boundaries of the domains $\Omega ^1_t$ and $\Omega ^2_t$ are sufficiently close, which will be the case of interest here, then $\Omega _t$ will have a Lipschitz boundary. Let $D_{t}^1$ and $D_{t}^2$ be the material derivatives associated with the domains $\Omega _t^1$ and $\Omega _t^2$, respectively. In $\Omega _t$ define the averaged material derivative

$$\begin{aligned} D_{t}{:=} \frac{D_{t}^1+D_{t}^2}{2} \end{aligned}$$

and the averaged $G$,

$$\begin{aligned} G^{ij}_{mid} {:=} G^{ij} \left( \frac{r_1+r_2}{2}, \frac{v_1+v_2}{2}\right) . \end{aligned}$$

We note that the above averaged material derivative is not exactly advecting the domain $\Omega _t$. Fortunately exact advection is not at all needed for what follows. See also Remark 4.3 below.

To measure the difference between two solutions on the common domain $\Omega _t$, we introduce the following distance functional:

4.1 $$\begin{aligned} {\mathcal {D}}_{{\mathcal H }}((r_1,v_1), (r_2,v_2)) {:=} \int _{\Omega _t} (r_1+r_2)^\frac{1-\kappa }{\kappa } ( (r_1-r_2)^2 +(r_1+r_2) |v_1-v_2|^2)\, dx \end{aligned}$$

which is the same as in [14]. We could have used $G$ to measure $|v_1-v_2|$, but the Euclidean metric suffices. This is not only because both metrics are comparable but also because we will not use (4.1) directly in conjunction with the equations. We will, however, use $G$ further below when we introduce another functional for which the structure of the equations will be relevant.

We observe the following Lemma, which has been proved in [14]:

### Lemma 4.1

Assume that $r_1$ and $r_2$ are uniformly Lipschitz and nondegenerate, and close in the Lipschitz topology. Then we have

4.2 $$\begin{aligned} \int _{\Gamma _t} |r_1+r_2|^{\frac{1}{\kappa } +2} d\sigma \lesssim {\mathcal {D}}_{\mathcal H }((r_1,v_1),(r_2,v_2)) . \end{aligned}$$

One can view the integral in (4.2) as a measure of the distance between the two boundaries, with the same scaling as ${\mathcal {D}}_{\mathcal H }$.

We now state our main estimate for differences of solutions:

### Theorem 4.2

Let $(r_1,v_1)$ and $(r_2,v_2)$ be two solutions for the system (1.16) in [0, *T*], with regularity $\nabla r_j \in \tilde{C}^\frac{1}{2}$, $v_j \in C^1$, so that $r_j$ are uniformly nondegenerate near the boundary and close in the Lipschitz topology, $j=1,2$. Then we have the uniform difference bound

4.3 $$\begin{aligned} \sup _{t \in [0,T]} {\mathcal {D}}_{\mathcal H }((r_1,v_1)(t),(r_2,v_2)(t)) \lesssim {\mathcal {D}}_{\mathcal H }((r_1,v_1)(0),(r_2,v_2)(0)). \end{aligned}$$

We remark that

$$\begin{aligned} {\mathcal {D}}_{\mathcal H }((r_1,v_1),(r_2,v_2)) = 0 \quad \text {iff} \quad (r_1,v_1)= (r_2,v_2), \end{aligned}$$

which implies our uniqueness result.

The remaining of this Section is dedicated to proving Theorem 4.2.

### A degenerate energy functional

We will not work directly with the functional $D_{\mathcal H }$ because it is non-degenerate, so we cannot take full advantage of integration by parts when we compute its time derivative. We thus consider the modified difference functional

4.4 $$\begin{aligned} {\tilde{\mathcal {D}}}_{\mathcal H }((r_1,v_1),(r_2,v_2)):= & {} \int _{\Omega _t} (r_1+r_2)^{\frac{1-\kappa }{\kappa }} \big ( a (r_1-r_2)^2\nonumber \\&+ b(a_{21}+a_{22})^{-1} G^{ij}_{mid} (v_1-v_2)_i (v_1-v_2)_j \big )\, dx,\nonumber \\ \end{aligned}$$

where $a_{21}$ and $a_{22}$ are the coefficient $a_2$ corresponding to the solutions $(r_1,v_1)$ and $(r_2,v_2)$, respectively, and *a* and *b* are functions of $\mu {:=} r_1+r_2$ and $\nu =r_1-r_2$ with the following properties

1. They are smooth, nonnegative functions in the region $\{ 0 \le |\nu | < \mu \}$, even in $\nu$, and homogeneous of degree 0, respectively 1.
2. They are connected by the relation $\mu a = b$.
3. They are supported in $\{ |\nu | < \frac{1}{2} \mu \}$, with $a = 1$ in $\{|\nu | < \frac{1}{4} \mu \}$.

#### Remark 4.3

The choice of the weights *a* and *b* above guarantees that the integrand in (4.4) above vanishes polynomially on the boundary of the common domain $\Omega _t$. This is why we refer to this difference functional as degenerate, and is also the reason why we are able to use the averaged material derivative $D_t$ to propagate bounds for ${\tilde{\mathcal {D}}}_{\mathcal H }$ in time even though it does not exactly advect $\Omega _t$.

From [14] we also borrow the equivalence property of the two distance functionals defined above:

#### Lemma 4.4

Assume that $A = A_1+A_2$ is small. Then

4.5 $$\begin{aligned} {\mathcal {D}}_{\mathcal H }((r_1,v_1),(r_2,v_2)) \approx _A {\tilde{\mathcal {D}}}_{\mathcal H }((r_1,v_1),(r_2,v_2)) . \end{aligned}$$

### The energy estimate

To prove the Theorem 4.2 it remains to track the time evolution of the degenerate distance functional ${\tilde{\mathcal {D}}}_{{\mathcal H }}$. This is the content of the next result, which immediately implies Theorem 4.2 after an application of Gronwall’s inequality.

#### Proposition 4.5

We have

4.6 $$\begin{aligned} \frac{d}{dt} {\tilde{\mathcal {D}}}_{\mathcal H }((r_1,v_1),(r_2,v_2)) \lesssim (B_1+B_2) {\mathcal {D}}_{\mathcal H }((r_1,v_1),(r_2,v_2)). \end{aligned}$$

#### Proof

The difference of the two solutions to (1.16) in the common domain $\Omega _t$ satisfies

4.7 $$\begin{aligned} \begin{aligned} 2 D_{t}(r_1 - r_2) =&- (D_{t}^1 - D_{t}^2) (r_1 + r_2) - (r_1 (G_1)^{ij} + r_2 (G_2)^{ij} )\partial _i ( (v_1)_j - (v_2)_j)\\&- (r_1 (G_1)^{ij} - r_2 (G_2)^{ij} )\partial _i ( (v_1)_j + (v_2)_j )\\&- (r_1 a_{11} v_1^i + r_2 a_{12} v_2^i )\partial _i (r_1 - r_2)\\&- (r_1 a_{11} v_1^i - r_2 a_{12} v_2^i )\partial _i (r_1 + r_2) , \end{aligned} \end{aligned}$$

and

4.8 $$\begin{aligned} \begin{aligned} 2D_{t}( (v_1)_i - (v_2)_i ) =&- (D_{t}^1 - D_{t}^2 )( (v_1)_i + (v_2)_i ) - (a_{2,1} + a_{2,2}) \partial _i (r_1 - r_2)\\&- (a_{21} - a_{22}) \partial _i (r_1 + r_2). \end{aligned} \end{aligned}$$

Above, $G_i$ and $a_{1i}$ correspond to $G$ and $a_1$ for the solutions $(r_i,v_i)$, $i=1,2$. We observe that the difference of material derivatives can be written as

$$\begin{aligned} (D_{t}^1 - D_{t}^2 ) = \left( \tilde{v}_1 - \tilde{v}_2 \right) \cdot \nabla , \qquad \tilde{v}^i = \frac{v^i}{v^0}. \end{aligned}$$

To compute the time derivative of the degenerate distance we use the standard formula for differentiation in a moving domain $\Omega _t$,

4.9 $$\begin{aligned} \frac{d}{dt}\int _{\Omega _t} f(t, x)\,dx= \int _{\Omega _t} D_tf + f \nabla \cdot v (t)\, dx, \end{aligned}$$

where *v* is in our case the average velocity. Classically this holds under the assumption that the domain $\Omega _t$ is advected by $D_t$. But in our case we replace this assumption with the alternative condition that *f* vanishes on the boundary of $\Omega _t$. Using this formula we obtain

$$\begin{aligned} \begin{aligned} \frac{d}{dt}{\tilde{\mathcal {D}}}_{{\mathcal H }}(t)&=I_1+I_2+I_3+I_4+I_5 +I_6+O(B_1+B_2)D_{{\mathcal H }}(t), \end{aligned} \end{aligned}$$

where the integrals $I_i$, $i=\overline{1,6}$, represent contributions as follows:

(a) $I_1$ represents the contribution where the averaged material derivative falls on *a* or *b*,

$$\begin{aligned} \begin{aligned} I_1=&\int _{\Omega _t} (r_1+r_2)^\frac{1-\kappa }{\kappa } [a_\mu (r_1-r_2)^2 + b_\mu (a_{21}+a_{22})^{-1} G^{ij}_{mid} (v_1-v_2)^2\,] D_t(r_1+r_2) \, dx\\&+ \int _{\Omega _t}(r_1+r_2)^\frac{1-\kappa }{\kappa } [a_\nu (r_1-r_2)^2 + b_\nu (a_{21}+a_{22})^{-1} G^{ij}_{mid} (v_1-v_2)^2\,] D_t(r_1-r_2) \, dx. \end{aligned} \end{aligned}$$

Here the derivatives of *a* and *b* are homogeneous of order $-1$, respectively 0. We get Gronwall terms when they get coupled with factors of $r_1+r_2$ or $r_1-r_2$ from the material derivatives. We discuss $I_1$ later.

(b) $I_2$ gathers the contributions where the averaged material derivative is applied to $(a_{21}+a_{22})^{-1}$ and to $G^{ij}_{mid}$. These expressions are smooth functions of $(r_1, v_1), (r_2,v_2)$, and thus their derivatives are bounded by $B_1+B_2$,

$$\begin{aligned} I_2=O(B_1+B_2)D_{{\mathcal H }}(t). \end{aligned}$$

(c) $I_3$ represents the main contribution of the averaged material derivative that falls onto $(r_1-r_2)$ respectively on $v_1-v_2$ which consists of the first and second terms on the RHS in (4.7), and the second term in (4.8):

$$\begin{aligned} \begin{aligned} I_3=&-\int _{\Omega _t} (r_1+r_2)^\frac{1-\kappa }{\kappa } a(r_1-r_2)\big [ (\tilde{v}^i_1 - \tilde{v}^i_2) \partial _i (r_1 + r_2)\\&\ \ \ + (r_1 (G_1)^{ij} + r_2 (G_2)^{ij} )\partial _i ( (v_1)_j - (v_2)_j )\big ]\, dx\\&- \int _{\Omega _t} (r_1+r_2)^\frac{1-\kappa }{\kappa } b G^{ij}_{mid} ((v_1)_j-(v_2)_j) \partial _i (r_1 - r_2)\, dx . \end{aligned} \end{aligned}$$

This term will need further discussion.

(d) In $I_4$ we place the contribution of the forth term on the RHS of (4.7):

$$\begin{aligned} \begin{aligned} I_4= -\int _{\Omega _t} (r_1+r_2)^\frac{1-\kappa }{\kappa } a(r_1-r_2)(r_1 a_{11} v_1^i + r_2 a_{12} v_2^i )\partial _i (r_1 - r_2)\, dx. \end{aligned} \end{aligned}$$

This term will be discussed later.

(e) $I_5$ is given by the third and fifth terms on the RHS in (4.7) and the third term on the RHS from (4.8)

$$\begin{aligned} \begin{aligned} I_5 =&\int _{\Omega _t} (r_1+r_2)^\frac{1-\kappa }{\kappa } a(r_1-r_2) (r_1 (G_1)^{ij} - r_2 (G_2)^{ij} )\partial _i ( (v_1)_j + (v_2)_j ) \, dx\\&-\int _{\Omega _t} (r_1+r_2)^\frac{1-\kappa }{\kappa } a(r_1-r_2) (r_1 a_{11} v_1^i - r_2 a_{12} v_2^i )\partial _i (r_1 + r_2) \, dx\\&-\int _{\Omega _t} (r_1+r_2)^\frac{1-\kappa }{\kappa } b (a_{21}+a_{22})^{-1}G^{ij}_{mid} ((v_1)_j-(v_2)_j)(a_{21} - a_{22}) \partial _i (r_1 + r_2)\, dx. \end{aligned} \end{aligned}$$

All of the terms in $I_5$ are straightforward Gronwall terms.

(f) $I_6$ contains the terms where $D_{t}$ falls on $(r_1+r_2)^\frac{1-\kappa }{\kappa }$:

$$\begin{aligned} \begin{aligned} I_6&= \frac{1-\kappa }{\kappa } \! \int _{\Omega _t} \! (r_1+r_2)^{\frac{1}{\kappa }-2} \left( a (r_1-r_2)^2\! +\! b(a_{21}\!+\!a_{22})^{-1} \right. \\&\quad \left. \times G^{ij}_{mid} (v_1-v_2)_i (v_1-v_2)_j \right) D_{t}(r_1+r_2)\, dx. \end{aligned} \end{aligned}$$

We will analyze $I_6$ later.

It remains to take a closer look at the integrals $I_1$, $I_3$, $I_4$, and $I_6$. We consider them in succession.

*The bound for* $I_1$. Here we write

$$\begin{aligned} 2 D_t (r_1+r_2) = 2 D_t^1 r_1 + D_t^2 r_2 - (\tilde{v}_1 - \tilde{v}_2) \cdot \nabla (r_1-r_2), \end{aligned}$$

and

$$\begin{aligned} 2 D_t (r_1-r_2) = 2 D_t^1 r_1 - D_t^2 r_2 - (\tilde{v}_1 - \tilde{v}_2)\cdot \nabla (r_1+ r_2) . \end{aligned}$$

The first two terms have size $O(B(r_1+r_2))$ so their contribution is a Gronwall term. We are left with the contribution of the last terms, which yields the expressions

$$\begin{aligned} \begin{aligned} I_1^a=&\int _{\Omega _t} (r_1+r_2)^\frac{1-\kappa }{\kappa } a_\mu (r_1-r_2)^2 (\tilde{v}_1 - \tilde{v}_2)\cdot \nabla (r_1-r_2) \, dx\\&+\! \int _{\Omega _t} \! (r_1+r_2)^\frac{1-\kappa }{\kappa } b_\mu (a_{21}+a_{22})^{-1} G^{ij}_{mid} ((v_1)_i-(v_2)_i)((v_1)_j\\&\quad -(v_2)_j) (\tilde{v}_1 - \tilde{v}_2)\!\cdot \! \nabla (r_1-r_2) \, dx,\\ I_1^b =&\int _{\Omega _t} (r_1+r_2)^\frac{1-\kappa }{\kappa } a_\nu (r_1-r_2)^2 (\tilde{v}_1 - \tilde{v}_2)\!\cdot \! \nabla (r_1+ r_2)\,dx\\&+ \! \int _{\Omega _t} \! (r_1+r_2)^\frac{1-\kappa }{\kappa } b_\nu (a_{21}+a_{22})^{-1} G^{ij}_{mid} ((v_1)_i-(v_2)_i)((v_1)_j\\ {}&\quad -(v_2)_j) (\tilde{v}_1 - \tilde{v}_2)\!\cdot \! \nabla (r_1+ r_2)\, dx . \end{aligned} \end{aligned}$$

For the second integral in both expressions, we bound $|\tilde{v}_1 - \tilde{v}_2| \lesssim |r_1-r_2|+|v_1-v_2|$ and estimate their part by

$$\begin{aligned} \int _{\Omega _t} (r_1+r_2)^\frac{1-\kappa }{\kappa } |r_1-r_2||v_1-v_2|^2 \, dx \lesssim D_{\mathcal H }(t) \end{aligned}$$

and

$$\begin{aligned} J_2 = \int _{\Omega _t} (r_1+r_2)^\frac{1-\kappa }{\kappa } |v_1-v_2|^3 \, dx, \end{aligned}$$

which is discussed later.

We are left with the first integrals in $I_1^a$ and $I_1^b$, which we record as

$$\begin{aligned} J_1^a=&\int _{\Omega _t} (r_1+r_2)^\frac{1-\kappa }{\kappa } a_\mu (r_1-r_2)^2 (\tilde{v}_1 - \tilde{v}_2)\cdot \nabla (r_1-r_2) \, dx \end{aligned}$$

and

$$\begin{aligned} J_1^b =&\int _{\Omega _t} (r_1+r_2)^\frac{1-\kappa }{\kappa } a_\nu (r_1-r_2)^2 (\tilde{v}_1 - \tilde{v}_2)\cdot \nabla (r_1+ r_2)\, dx. \end{aligned}$$

These integrals are also discussed later.

*The bound for* $I_3$. For $I_3$, we seek to capture the same cancellation that it is seen in the analysis of the linearized equation. We look at the last term in $I_3$, use $b=a(r_1+r_2)$, and integrate by parts; if the derivatives falls on $G$ then this is a straightforward Gronwall term. We are left with three contributions, two of which we pair with the first two terms in $I_3$. We obtain

$$\begin{aligned} \begin{aligned} I_3=&-\int _{\Omega _t} (r_1+r_2)^\frac{1-\kappa }{\kappa } a(r_1-r_2)\left[ (\tilde{v}^i_1 - \tilde{v}^i_2) -\frac{1}{\kappa } G^{ij}_{mid} ((v_{1})_j- (v_{2})_j )\right] \partial _i (r_1 + r_2) \, dx \\&-\int _{\Omega _t} (r_1+r_2)^\frac{1-\kappa }{\kappa } a(r_1-r_2) \left[ (r_1 (G_1)^{ij} + r_2 (G_2)^{ij} ) - (r_1+r_2)G^{ij}_{mid}\right] \partial _i ( (v_1)_j - (v_2)_j )\, dx\\&+\int _{\Omega _t} (r_1+r_2)^\frac{1}{\kappa } \partial _i a G^{ij}_{mid} ((v_1)_j-(v_2)_j) (r_1 - r_2)\, dx +O(B_1+B_2) D_{{\mathcal H }}\\&= I_3^1+I_3^2+ J_1^c +O(B_1+B_2) D_{{\mathcal H }} . \end{aligned} \end{aligned}$$

For the first integral, $I_3^1$ we expand the difference $\tilde{v}^i_1 - \tilde{v}^i_2$, seen as a function of $v_1$ and $v_2$, in a Taylor series around the center $(v_1+v_2)/2$. We have

$$\begin{aligned} \dfrac{\partial \tilde{v}_i}{\partial v_j} = \frac{1}{v^0} \left( \delta ^{ij}-\frac{v_i v_j}{(v^0)^2}\right) , \end{aligned}$$

where we recognize the matrix on the right as being the main part in $G$. Thus, we can write

$$\begin{aligned} \left| (\tilde{v}^i_1 - \tilde{v}^i_2) - \frac{1}{\kappa } G^{ij}_{mid} ((v_{1})_j-(v_{2})_j) \right| \lesssim \vert r_1-r_2\vert + (r_1+r_2) \vert v_1-v_2\vert + \vert v_1-v_2\vert ^3, \end{aligned}$$

where the quadratic $v_1-v_2$ terms cancel because we are expanding around the middle, and we used (3.2) to get the second term on the right. The contributions of all of the terms in the last expansion are Gronwall terms.

For the second integral $I_3^2$ we have a simpler expansion

$$\begin{aligned} \left| (r_1 (G_1)^{ij} + r_2 (G_2)^{ij} ) - (r_1+r_2)G^{ij}_{mid}\right| \lesssim \vert r_1-r_2\vert + (r_1+r_2)\vert (v_{1})_j-(v_{2})_j\vert ^2, \end{aligned}$$

where all contributions qualify again as Gronwall terms.

Finally, the last integral, $J_1^c$, is estimated below.

*The bound for* $I_4$. After an integration by parts we have

$$\begin{aligned} \begin{aligned} I_4&= \frac{1}{2}\int _{\Omega _t} (r_1+r_2)^\frac{1-\kappa }{\kappa } \partial _i a \, (r_1 a_{11} v_1^i + r_2 a_{12} v_2^i )(r_1 - r_2)^2\, dx\\&\quad + \frac{1-\kappa }{2\kappa } \int _{\Omega _t} (r_1+r_2)^{\frac{1}{\kappa } - 2} \partial _i (r_1+r_2) a(r_1 a_{11} v_1^i + r_2 a_{12} v_2^i )(r_1 - r_2)^2\, dx\\&\quad + O(B_1+B_2) D_{{\mathcal H }}. \end{aligned} \end{aligned}$$

Writing

$$\begin{aligned} |r_1 a_{11} v_1^i + r_2 a_{12} v_2^i|&\lesssim r_1+r_2, \end{aligned}$$

both integrals are bounded by $O(B_1+B_2) D_{{\mathcal H }}$.

*The bound for* $I_6$. We use $D_{t}(r_1+r_2) = D_t^1 r_1 + D_t^2 r_2 - (\tilde{v}_1 - \tilde{v}_2) \cdot \nabla ( r_1-r_2)$ where the first two terms are bounded by $(B_1+B_2)(r_1+r_2)$ and yield Gronwall contributions. Then we write

$$\begin{aligned} I_6 \lesssim J_1^d + J_2 + O(B_1+B_2) D_{\mathcal H }, \end{aligned}$$

where

$$\begin{aligned} \begin{aligned} J_1^d&= \frac{1-\kappa }{\kappa } \int _{\Omega _t} (r_1+r_2)^{\frac{1}{\kappa }-2} a (r_1-r_2)^2 (\tilde{v}_1 - \tilde{v}_2) \cdot \nabla ( r_1-r_2)\, dx. \end{aligned} \end{aligned}$$

To summarize the outcome of our analysis so far, we have proved that

$$\begin{aligned} \frac{d}{dt}\tilde{D}_{{\mathcal H }}(t) \le J_1^a + J_1^b + J_1^c + J_1^d + O(J_2) + O(B_1+B_2) D_{\mathcal H }. \end{aligned}$$

It remains to estimate $J_2$, $J_1^a$, $J_1^b$, $J_1^c$, and $J_1^d$, which we write here again for convenience:

$$\begin{aligned} \begin{aligned} J_2&= \int _{\Omega _t} (r_1+r_2)^\frac{1-\kappa }{\kappa } |v_1-v_2|^3 \, dx, \\ J_1^a&= \int _{\Omega _t} (r_1+r_2)^\frac{1-\kappa }{\kappa } a_\mu (r_1-r_2)^2 (\tilde{v}_1 - \tilde{v}_2)\cdot \nabla (r_1-r_2) \, dx, \\ J_1^b&= \int _{\Omega _t} (r_1+r_2)^\frac{1-\kappa }{\kappa } a_\nu (r_1-r_2)^2 (\tilde{v}_1 - \tilde{v}_2)\cdot \nabla (r_1+ r_2)\, dx, \\ J_1^c&= \int _{\Omega _t} (r_1+r_2)^\frac{1}{\kappa } \partial _i a G^{ij}_{mid} ((v_1)_j-(v_2)_j) (r_1 - r_2)\, dx, \\ J_1^d&= \frac{1-\kappa }{\kappa } \int _{\Omega _t} (r_1+r_2)^{\frac{1}{\kappa }-2} a (r_1-r_2)^2 (\tilde{v}_1 - \tilde{v}_2) \cdot \nabla ( r_1-r_2)\, dx. \end{aligned} \end{aligned}$$

The integral $J_2$ is the same as in [14] and can be estimated accordingly, using interpolation inequalities; see Lemma 4.4 in [14].

The bound for the integrals $J_1^a$, $J_1^b$, $J_1^c$ and $J_1^d$ matches estimates for similar integrals in [14]. Precisely, the integrals $J_1^a$ and $J_2^b$ are estimated as the integrals called $J_1^b$ and $J_1^c$ in [14], respectively, see Lemmas 4.6–4.13 in [14]. The integral $J_1^c$ is estimated as the integral $J_1^d$ in [14], see Lemmas 4.6–4.13 in [14]. The integral $J_1^d$ is estimated as the integral $J_1^a$ in [14], see Lemmas 4.6–4.13 in [14].

We caution the reader that the arguments in [14] are not straightforward, and involve peeling off a carefully chosen boundary layer, with separate estimates inside the boundary layer and outside it. The only difference in the present paper is the presence of additional weights in the integrals, which are smooth functions of $r_1,r_2,v_1,v_2$. For instance, the difference $\tilde{v}_1 - \tilde{v}_2$ can be expanded as

$$\begin{aligned} \tilde{v}_1 - \tilde{v}_2 = f(r,v) (r_1-r_2) + g(r,v) (v_1-v_2), \end{aligned}$$

where *r*, *v* stand for $r_1,r_2,v_1,v_2$ and *f*, *g* are smooth. The contribution of the first term admits a straightforward Gronwall bound, and the contribution of the second term is akin to the corresponding integral in [14] but with the added smooth weight. The point is that every time we integrate by parts and the derivative falls on the smooth weight, the corresponding contribution is a straightforward Gronwall term. Hence such smooth weights make no difference if added in the arguments in [14].

$$\square $$

## Energy estimates for solutions

Our goal in this section is to establish uniform control over the $\mathbf {H}^{2k}$ norm of the solutions (*r*, *v*) to (1.16) with growth given by the norms *A* and *B*. For this, we will use appropriate energy functionals $E^{2k}= E^{2k}(r,v)$ constructed out of vector fields naturally associated with the evolution. Our functionals will be associated with the wave and transport parts of the system, which will be considered at matched regularity.

The vector fields we will consider are:

- The material derivative $D_{t}$, which has order 1/2.
- All regular derivatives $\partial$, which have order 1.
- Multiplication by *r*, which has order $-1$.

The wave part of the energy will be associated mainly with $D_{t}$, whereas the transport part will be associated with all of the above vector fields.

### Good variables and the energy functional

Heuristically, higher order energy functionals should be obtained by applying an appropriate number of vector fields to the equation, and then verifying that the output solves the linearized equation modulo perturbative terms. In the absence of the free boundary, there are two equally good choices, (i) to spatially differentiate the equation, using the $\partial _j$ vector fields, or (ii) to differentiate the equation in time, using the $\partial _t$ vector field.

However, in the free boundary setting, both of the above choices have issues, as neither $\partial _j$ nor $\partial _t$ are adapted to the boundary. For $\partial _t$ we do have a seemingly better choice, namely to replace it by the material derivative $D_t$. However, this has the downside that it does not arise from a symmetry of the equations, and consequently the expressions $(D_t^{2k} r, D_t^{2k} v)$ are not good approximate solutions to the linearized equations. To address this matter, we add suitable corrections to these expressions, obtaining what we call the *good variables* $(s_{2k},w_{2k})$. Precisely, motivated by the linearized equations (3.1), we introduce

5.1 $$\begin{aligned} \begin{aligned} s_0 {:=} r,\\ w_0 {:=} v,\\ s_1 {:=} \partial _t r,\\ w_1 {:=} \partial _t v,\\ s_2 {:=}D_{t}^2r + \frac{1}{2} \frac{a_0 a_2}{\kappa \langle r \rangle } G^{ij} \partial _i r \partial _j r, \\ (w_k)_i {:=} D_{t}^k v_i, \, \qquad k \ge 2, \\ s_k {:=} D_{t}^k r - \frac{a_0}{\kappa \langle r \rangle } G^{ij} \partial _i r D_{t}^{k-1} v_j, \qquad \, k\ge 3. \end{aligned} \end{aligned}$$

Here, we use the full Eq. (1.16) to interpret $(s_j,w_j)$ as multilinear expressions in (*r*, *v*), with coefficients which are functions of undifferentiated (*r*, *v*). Observe that it would be compatible with the linearized equations to define $s_k$ with $\dfrac{1}{\kappa } G^{ij} \partial _i r D_{t}^{k-1} v_j$ instead of $\dfrac{a_0}{\kappa \langle r \rangle } G^{ij} \partial _i r D_{t}^{k-1} v_j$. The difference between the two cases, however, is a perturbative term due to (3.2), and the definition we adopt here is more convenient because $\dfrac{a_0}{\kappa \langle r \rangle }$ is what appears in the commutator $[D_{t},\partial ]$.

Using equations (1.16), we find that for $k \ge 1$, our good variables $(s_{2k}, w_{2k})$ can be seen as approximate solutions to the linearized equation (3.1). Precisely, they satisfy the following equations in $\Omega$ (compare with (3.1)):
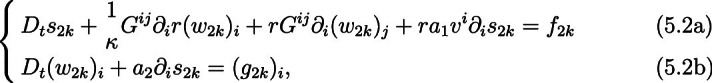


with source terms $(f_{2k},g_{2k})$ which will be shown to be perturbative, see Lemma 5.7. For later use we compute the expressions for the source terms $(f_{2k},g_{2k})$, which are given by

5.3a $$\begin{aligned} f_{2k}= & {} [ r G^{ij} \partial _i, D_{t}^{2k} ] v_j + [r a_1 v^i \partial _i, D_{t}^{2k} ] r - r \frac{a_0 a_1}{\kappa \langle r \rangle } G^{pq} \partial _q r v^i \partial _i D_{t}^{2k-1} v_p \nonumber \\ \end{aligned}$$

5.3b $$\begin{aligned}&- D_{t}\left( \frac{a_0}{\kappa \langle r \rangle } G^{ij} \partial _i r \right) D_{t}^{2k-1} v_j - r a_ 1 v^i \partial _i \left( \frac{a_0}{\kappa \langle r \rangle } G^{pq} \partial _p r \right) D_{t}^{2k-1} v_q \nonumber \\&- r a_3 G^{ij} \partial _i r (w_{2k})_j , \nonumber \\ (g_{2k})_i= & {} D_{t}^{2k-1} [ a_2 \partial _i, D_{t}] r + [a_2 \partial _i, D_{t}^{2k-1} ] D_{t}r - \frac{a_0 a_2}{\kappa \langle r \rangle }G^{jl} \partial _j r \partial _i D_{t}^{2k-1} v_l \nonumber \\&-a_2 \partial _i\left( \frac{a_0}{\kappa \langle r \rangle } G^{ml} \partial _m r \right) D_{t}^{2k-1} v_l, \end{aligned}$$

where we used that

$$\begin{aligned}{}[A,BC] = [A,B]C + B[A,C]. \end{aligned}$$

to write

$$\begin{aligned}{}[a_2 \partial _i, D_{t}^{2k} ] = [a_2 \partial _i, D_{t}^{2k-1}] D_{t}+ D_{t}^{2k-1} [ a_2 \partial _i, D_{t}]. \end{aligned}$$

We also define

5.4 $$\begin{aligned} \omega _{2k} = r^a \partial ^b \omega , \, |b| \le 2k-1, \, |b|-a = k-1 , \end{aligned}$$

which we think of as the vorticity counterpart to $(s_{2k},w_{2k})$. These we will think of as solving approximate transport equations; using (1.22) we find

5.5 $$\begin{aligned} D_{t}(\omega _{2k})_{ij}&+ \frac{1}{v^0} (\partial _i v^l (\omega _{2k})_{l j} + \partial _j v^l (\omega _{2k})_{i l } ) - \frac{v^l}{(v^0)^2} (\partial _iv^0v (\omega _{2k})_{lj} - \partial _j v^0 (\omega _{2k})_{li})\nonumber \\&= (h_{2k})_{ij}, \end{aligned}$$

where $h_{2k}$ is given by

5.6 $$\begin{aligned} (h_{2k})_{ij}&= \left[ D_{t}, r^a \partial ^b\right] \omega _{ij} + \left[ \frac{1}{v^0} \partial _i v^l , r^a \partial ^b\right] \omega _{l j} + \left[ \frac{1}{v^0} \partial _j v^l,r^a\partial ^b\right] \omega _{i l }\nonumber \\&\quad - \left[ \frac{1}{(v^0)^2} \partial _iv^0v^l,r^a \partial ^b\right] \omega _{lj} + \left[ \frac{1}{(v^0)^2} \partial _j v^0 v^l,r^a\partial ^b\right] \omega _{li}. \end{aligned}$$

We introduce the wave energy

$$\begin{aligned} E^{2k}_{\text {wave}}(r,v) {:=} \sum _{j=0}^{k} \Vert (s_{2j}, w_{2j}) \Vert ^2_{\mathcal {H}}, \end{aligned}$$

the transport energy

$$\begin{aligned} E^{2k}_{\text {transport}}(r,v) {:=} \Vert \omega \Vert ^2_{H^{2k-1,k+ \frac{1}{2\kappa }}}, \end{aligned}$$

and the total energy

5.7 $$\begin{aligned} E^{2k}(r,v) {:=} E^{2k}_{\text {wave}}(r,v) + E^{2k}_{\text {transport}}(r,v) . \end{aligned}$$

### Energy coercivity

Our goal in this section is to show that the energy (5.7) measures the $\mathbf {H}^{2k}$ size of (*r*, *v*). To do so, we would like to consider the energy as a functional of (*r*, *v*) defined at a fixed time. This can be done by using Eq. (1.16) to algebraically solve for spatial derivatives of (*r*, *v*).

#### Theorem 5.1

Let (*r*, *v*) be smooth functions in $\overline{\Omega }$. Assume that *r* is positive in $\Omega$ and uniformly non-degenerate on the $\Gamma$. Then

$$\begin{aligned} E^{2k} \approx _A \Vert (r,v) \Vert ^2_{\mathcal {H}^{2k}}. \end{aligned}$$

#### Proof

We begin with the $\lesssim$ part. We consider the wave part of the energy and the corresponding expressions for $(s_{2k},w_{2k})$. Using use Eq. (1.16a) and (1.16b) to successively solve for $D_{t}(r,v)$, we obtain that each $(s_{2k},w_{2k})$ is a linear combination of multilinear expressions in *r* and $\partial v$ (with order zero coefficients).

We will use our bookkeeping scheme of Section 1.3 to understand the expressions for $(s_{2k},w_{2k})$. It is useful to record here the order and structure of the linear-in-derivatives top order terms obtained by using the equations to inductively compute $D_{t}^{2k} (r,v)$ and $D_{t}^{2k-1}(r,v)$, which involve 2*k* and $2k-1$ derivatives, respectively:

5.8a $$\begin{aligned} D_{t}^{2k} r&\approx r^k \partial ^{2k}r + r^{k+1} \partial ^{2k} v \approx r^k \partial ^{2k} r, \end{aligned}$$

5.8b $$\begin{aligned} (k-1)&\approx (k-1) + (k-\frac{3}{2}) \approx (k-1), \nonumber \\ D_{t}^{2k} v&\approx r^k \partial ^{2k} v + r^{k} \partial ^{2k} r \approx r^k \partial ^{2k} v , \end{aligned}$$

5.8c $$\begin{aligned} (k-\frac{1}{2})&\approx (k-\frac{1}{2}) + (k-1) \approx (k-\frac{1}{2}) , \nonumber \\ D_{t}^{2k-1} r&\approx r^{k} \partial ^{2k-1}r + r^{k} \partial ^{2k-1} v \approx r^{k} \partial ^{2k-1} v, \end{aligned}$$

5.8d $$\begin{aligned} (k-\frac{3}{2})&\approx (k-2) + (k-\frac{3}{2}) \approx (k-\frac{3}{2}), \nonumber \\ D_{t}^{2k-1} v&\approx r^{k} \partial ^{2k-1} v + r^{k-1} \partial ^{2k-1} r \approx r^{k-1} \partial ^{2k-1} r, \\ (k -1 )&\approx (k-\frac{3}{2}) + (k-1) \approx (k-1). \nonumber \end{aligned}$$

Expressions (5.8) are obtained by successively solving for $D_{t}(r,v)$ in (1.16a)–(1.16b). Below each expression in (5.8a)-(5.8d) we have written the orders of the corresponding terms. The terms of order $k-3/2$, $k-1$, $k-2$, and $k-3/2$ in (5.8a), (5.8b), (5.8c), and (5.8d), respectively have orders less than the other terms in the same expressions, despite having the same number of derivatives, and hence are dropped in the second $\approx$ on each line. Such terms have smaller order, even though they have the same number of derivatives, because of extra powers of *r*, and come from the term $r a_1 v^i \partial _i r$ in (1.16a).

We begin with the expressions of highest order (see Section 1.3), thus we first focus on the multilinear expressions that come from ignoring the last term on LHS (1.16a) and also where no derivative lands on $G$, $a_1$, and $a_2$. We also consider first the case when every time we commute $D_{t}$ with $\partial$, the derivative lands on $v^i$ and not on *r* via $v^0$.

In this case, the corresponding multilinear expressions for $(s_{2k},w_{2k})$ have the following properties:

- They have order $k-1$ and $k-\frac{1}{2}$, respectively.
- They have exactly 2*k* derivatives.
- They contain at most $k+1$ and *k* factors of *r*, respectively.

Thus, a multilinear expression for $s_{2k}$ in this case has the form

5.9 $$\begin{aligned} M = r^a \prod _{j=1}^J \partial ^{n_j} r \prod _{l=1}^L \partial ^{m_l} v, \end{aligned}$$

where $n_j,m_l \ge 1$, and subject to

5.10 $$\begin{aligned} \begin{aligned}&\sum n_j + \sum m_l = 2k,\\&a + J + L/2 = k+1, \end{aligned} \end{aligned}$$

and when $J=0$ or $L=0$ the corresponding product is omitted. We claim that it is possible to choose $b_j$ and $c_l$ such that

$$\begin{aligned} 0 \le b_j \le (n_j - 1 ) \frac{k}{2k-1}, \, 0 \le c_l \le (m_l - 1) \frac{k+1/2}{k - 1/2}, \, a = \sum b_j + \sum c_l. \end{aligned}$$

This follows from observing that

$$\begin{aligned}&\sum (n_j - 1 ) \frac{k}{2k-1} + \sum (m_l - 1) \frac{k+1/2}{k - 1/2} \le \left( \sum n_j + \sum m_l - J - L \right) \frac{k}{2k-1} \\&\quad = (2k-J-L) \frac{k}{2k-1} \le (a+k-1) \frac{k}{2k-1} \le a, \end{aligned}$$

since $a\le k$. Equality holds only if $a=k$, $J=1$ and $L=0$ (i.e., for the leading linear case). This shows that it is possible to make such a choice of $b_j$ and $c_l$, which allows us to use our interpolation theorems

$$\begin{aligned} \Vert r^{b_j} \partial ^{n_j} r\Vert _{L^{p_j}(r^{\frac{1-\kappa }{\kappa }})}&\lesssim (1+A)^{1-\frac{2}{p_j}} \Vert (r,v)\Vert _{\mathcal {H}^{2k}}^{\frac{2}{p_j}}, \\ \Vert r^{c_l} \partial ^{m_l} v \Vert _{L^{q_l}(r^{\frac{1-\kappa }{\kappa }})}&\lesssim A^{1-\frac{2}{q_l}} \Vert (r,v)\Vert _{\mathcal {H}^{2k}}^{\frac{2}{q_l}}, \end{aligned}$$

where

$$\begin{aligned} \frac{1}{p_j} = \frac{n_j -1 -b_j}{2(k-1)}, \, \frac{1}{q_l} = \frac{m_l -1/2 -c_l}{2(k-1)}. \end{aligned}$$

Observe that the numerators in $1/p_j$ and $1/q_l$ correspond to the orders of the expressions being estimated and they add up to $k-1$ (as needed).

In addition to the principal part discussed above, we also obtain lower order terms in our expression for $s_{2k}$. There are three sources of such terms:

i) The terms from the commutator $[D_{t},\partial ]$ where derivatives apply to *r* via $v^0$. This corresponds to the second term in the formal expansion
  $$\begin{aligned}{}[D_{t}, \partial ] \approx (\partial v )\partial + (\partial r) \partial , \end{aligned}$$
  whose order is easily seen to be 1/2 lower.
ii) If any derivatives are applied to either *r* or *v* via $a_0$, $a_1$, $a_2$ or *G*, this increases the order of the resulting expression by 0, respectively 1/2, compared to the full order of the derivative which is 1.
iii) Contributions arising from the last term in (1.15), whose order is, to start with, 1/2 lower than the rest of the terms in the (1.15).

The contributions of all such terms to $s_{2k}$ have lower order. More precisely, they contain expressions of the form (5.9) but with (5.10) replaced by

5.11 $$\begin{aligned} \begin{aligned} \sum n_j + \sum m_l&= 2k,\\ a + J + L/2&= k+1+\frac{j}{2}, \end{aligned} \end{aligned}$$

where $j > 0$, and which have lower order $k-1-\frac{j}{2}$. All such lower order terms can be estimated in a similar fashion, but using lower Sobolev norms for (*r*, *v*).

We continue with the $\gtrsim$ part. Applying $D_{t}$ to (5.2a) and (5.2b) and using definitions (5.1) we find the following recurrence relations

5.12a $$\begin{aligned} s_{2j}&= \tilde{L}_1 s_{2j-2} + F_{2j}, \end{aligned}$$

5.12b $$\begin{aligned} (w_{2j})_i&= (\tilde{L}_2 w_{2j-2} )_i + G_{2j}, \end{aligned}$$

where $\tilde{L}_1$ and $\tilde{L}_2$ have been defined in Section 3. The next Lemma characterizes the error terms on the RHS of (5.12), and the lemma that follows gives a quantitative relation between the 2*j* and $2j-2$ quantities.

#### Lemma 5.2

For $j\ge 2$, the terms $F_{2j}$ and $G_{2j}$ in (5.12) are linear combinations of multilinear expressions in *r* and $\partial v$ with 2*j* derivatives and of order at most $j-1$ and $j-\frac{1}{2}$, respectively. Moreover, they are either

(i) non-endpoint, by which we mean multilinear expressions of order $j-1$ and $j-\frac{1}{2}$, respectively, containing at most $j+1$ and *j* factors of *r*, respectively, and whose products contain at least two factors of $\partial ^{\ge 2} r$ or $\partial ^{\ge 1} v$, or
(ii) they have order strictly less than $j-1$ and $j-\frac{1}{2}$, respectively, and contain at most $j+2$ and $j+1$ factors of *r*, respectively.

#### Proof

We begin with $j\ge 3$. We will analyze

5.13 $$\begin{aligned} s_{2j}&= D_{t}^{2j} r - \frac{a_0}{\kappa \langle r \rangle } G^{lm}\partial _l r D_{t}^{2j-1} v_m. \end{aligned}$$

In order to keep track of terms according to the statement of the Lemma, we observe that $s_{2j}$ has order $j-1$. We will make successive use of the commutator

$$\begin{aligned} {\begin{matrix} [D_{t},\partial _l] = -\frac{a_0}{\kappa \langle r \rangle } G^{pq} \partial _l v_q \partial _p + \frac{\langle r \rangle ^{1+\frac{2}{\kappa }}}{(v^0)^3} v^p \partial _l r \partial _p. \end{matrix}} \end{aligned}$$

We begin with the first term on RHS (5.13). From (1.16), we compute.

$$\begin{aligned} D_{t}^2 r&= rG^{ml} \partial _l (a_2 \partial _m r) -[D_{t}, rG^{ml} \partial _l ] v_m - [D_{t}, r a_1 v^l \partial _l] r + r a_1 v^i \partial _i \left( rG^{ml} \right) \partial _l v_m\nonumber \\&\ \ \ + r a_1 v^i \partial _i (r a_1 v^l \partial _l )r + r^2 a_1^2 v^l v^m \partial _l \partial _m r + r^2 a_1 G^{ml} v^i \partial _i \partial _l v_m. \end{aligned}$$

Then,

5.14 $$\begin{aligned} D_{t}^{2j} r&= D_{t}^{2j-2} D_{t}^2 r = D_{t}^{2j-2} \Big ( rG^{ml} \partial _l (a_2 \partial _m r) \nonumber \\&-[D_{t}, rG^{ml} \partial _l ] v_m - [D_{t}, r a_1 v^l \partial _l]r \nonumber \\&+ r a_1 v^i \partial _i \left( rG^{ml} \right) \partial _l v_m + r a_1 v^i \partial _i (r a_1 v^l \partial _l )r \nonumber \\&+ r^2 a_1^2 v^l v^m \partial _l \partial _m r + r^2 a_1 G^{ml} v^i \partial _i \partial _l v_m, \Big ). \end{aligned}$$

and we will consider each term on RHS (5.14) separately.

The terms $\partial _i \left( rG^{ml} \right)$ and $\partial _i (r a_1 v^l \partial _l )r$ have order at most zero, thus

$$\begin{aligned} D_{t}^{2j-2} \left( r a_1 v^i \partial _i \left( rG^{ml} \right) \partial _l v_m + r a_1 v^i \partial _i (r a_1 v^l \partial _l )r \right) \end{aligned}$$

has order at most $j-3/2$ and belongs to $F_{2j}$. Next,

$$\begin{aligned} {\begin{matrix} [D_{t},rG^{ml} \partial _l] v_m = [D_{t},rG^{ml} ] \partial _l v_m + rG^{ml} [D_{t},\partial _l] v_m . \end{matrix}} \end{aligned}$$

For the first term on the RHS, we have

$$\begin{aligned}{}[D_{t},rG^{ml}] \partial _lv_m&= D_{t}r G^{lm} \partial _l v_m + r \frac{\partial G^{lm}}{\partial r} D_{t}r \partial _l v_m + r \frac{\partial G^{lm}}{\partial v_i} D_{t}v_i \partial _l v_m. \end{aligned}$$

The second and third terms have order $\le -1$ and $-1/2$, respectively, thus they belong to $F_{2j}$ after differentiation by $D_{t}^{2j-2}$. For the first term, we have

$$\begin{aligned} D_{t}r G^{lm} \partial _l v_m&= - r G^{ij} \partial _i v_j G^{lm} \partial _l v_m - r a_1 v^i \partial _i r G^{lm} \partial _l v_m. \end{aligned}$$

The first term satisfies the non-endpoint property while the second has order $-1/2$, thus both terms belong to $F_{2j}$ after differentiation by $D_{t}^{2j-2}$. Next,

$$\begin{aligned} r G^{ml} [D_{t}, \partial _l] v_m&= -\frac{ r a_0}{\kappa ( 1 + \langle r \rangle } G^{pq} \partial _l v_q G^{ml} \partial _p v_m + \frac{r \langle r \rangle ^{1+\frac{2}{\kappa }}}{(v^0)^3}G^{ml} v^p \partial _l r \partial _p v_m. \end{aligned}$$

The second term has order $-1/2$ so it belongs to $F_{2j}$ upon differentiation by $D_{t}^{2j-2}$. The first term has order zero, thus producing a top order (i.e., $j-1$) term when differentiated by $D_{t}^{2j-2}$. Nevertheless, it has two $\partial ^{\ge 1}v$ terms so it satisfies the non-endpoint property and hence it also belongs to $F_{2j}$.

We now turn to the other commutator in (5.14):

$$\begin{aligned}{}[D_{t}, r a_1 v^l \partial _l] r&= [D_{t}, r a_1 v^l] \partial _l r + r a_1 v^l [D_{t},\partial _l] r \nonumber \\&= D_{t}r a_1 v^l \partial _l r + r \frac{\partial a_1}{\partial r} D_{t}r v^l \partial _l r + r \frac{\partial a_1}{\partial v_i} D_{t}v_i v^l \partial _l r + r a_1 D_{t}v^l \partial _l r \nonumber \\&-\frac{r a_0 a_1 }{\kappa \langle r \rangle } G^{pq} v^l \partial _l v_q \partial _p r + \frac{r a_1 \langle r \rangle ^{1+\frac{2}{\kappa }}}{(v^0)^3} v^p v^l \partial _l r \partial _p r. \end{aligned}$$

The terms on the RHS have orders $\le -1/2,-3/2,-1,-1,-1/2,-1$, respectively, so they all belong to $F_{2j}$ upon differentiation by $D_{t}^{2j-2}$.

For the last two terms on RHS (5.14),

$$\begin{aligned} r^2 a_1^2 v^l v^m \partial _l \partial _m r + r^2 a_1 G^{ml} v^i \partial _i \partial _l v_m, \end{aligned}$$

we see that they have orders $-1$ and $-1/2$, thus also belong to $F_{2j}$ after differentiation by $D_{t}^{2j-2}$.

Therefore, writing $\approx$ for equality modulo terms that belong to $F_{2j}$, (5.14) becomes

$$\begin{aligned} \begin{aligned} D_{t}^{2j} r&= D_{t}^{2j-2} D_{t}^2 r\\&\approx D_{t}^{2j-2} \Big ( rG^{ml} \partial _l (a_2 \partial _m r) \Big ) \\&= \sum _{\ell =0}^{2j-2} \left( {\begin{array}{c}2j-2\\ \ell \end{array}}\right) D_{t}^{2j-2-\ell } r D_{t}^\ell \Big ( G^{ml} \partial _l (a_2 \partial _m r) \Big )\\&= r D_{t}^{2j-2} \Big ( G^{ml} \partial _l (a_2 \partial _m r) \Big ) + \sum _{\ell =0}^{2j-2-1} \left( {\begin{array}{c}2j-2\\ \ell \end{array}}\right) D_{t}^{2j-2-\ell } r D_{t}^\ell \Big ( G^{ml} \partial _l (a_2 \partial _m r) \Big ). \end{aligned} \end{aligned}$$

In the second sum, we can further write

$$\begin{aligned} D_{t}^{2j-2-\ell } r D_{t}^\ell \Big ( G^{ml} \partial _l (a_2 \partial _m r) \Big )&= D_{t}^{2j-2-\ell } r D_{t}^\ell \Big ( G^{ml} a_2 \partial _l \partial _m r + G^{ml} \partial _l a_2 \partial _m r \Big ) \end{aligned}$$

The term $D_{t}^{2j-2-\ell } r D_{t}^\ell ( G^{ml} \partial _l a_2 \partial _m r )$ has order at most $j-3/2$ and can be absorbed into $F_{2j}$. For the first term, if $D_{t}^\ell$ hits $G^{ml} a_2$ we again obtain a term of order strictly less than $j-1$ that is part of $F_{2j}$. Finally, for the term

$$\begin{aligned} D_{t}^{2j-2-\ell } r G^{ml} a_2 D_{t}^\ell \partial _l \partial _m r, \end{aligned}$$

we use that $\ell \le 2j-2-1$ and (1.16a) to write

$$\begin{aligned} D_{t}^{2j-2-\ell } r G^{ml} a_2 D_{t}^\ell \partial _l \partial _m r&= -G^{ml} a_2 D_{t}^\ell \partial _l \partial _m r D_{t}^{2j-3-\ell } \left( rG^{pq} \partial _p v_q \right) \nonumber \\&\ \ \ -G^{ml} a_2 D_{t}^\ell \partial _l \partial _m r D_{t}^{2j-3-\ell } \left( r a_1 v^p \partial _p r \right) . \end{aligned}$$

The first term contains a $\partial ^{\ge 1} v$ and a $\partial ^{\ge 2}r$ so it belongs to $F_{2j}$, whereas the second term has order at most $j-3/2$ so it belongs to $F_{2j}$ as well. Hence, we have that

$$\begin{aligned} D_{t}^{2j} r&\approx r D_{t}^{2j-2} \Big ( G^{ml} \partial _l (a_2 \partial _m r) \Big )\\&= rD_{t}^{2j-3} \Big ( G^{ml} \partial _l (a_2 \partial _m D_{t}r ) \Big ) + rD_{t}^{2j-3} \Big ( [D_{t}, G^{lm} \partial _l (a_2 \partial _m \cdot ) ] r \Big ). \end{aligned}$$

We now compute the commutator on the second term on the RHS:

$$\begin{aligned}{}[D_{t}, G^{lm} \partial _l (a_2 \partial _m \cdot ) ] r&= D_{t}\left( G^{lm} \partial _l (a_2 \partial _m r) \right) - G^{lm} \partial _l \left( a_2 \partial _m D_{t}r \right) \\&= \frac{\partial G^{lm}}{\partial r}D_{t}r \partial _l(a_2\partial _m r) + \frac{\partial G^{lm}}{\partial v_i}D_{t}v_i \partial _l(a_2\partial _m r) \\&\ \ \ + G^{lm} D_{t}\partial _l (a_2 \partial _m r) - G^{lm} \partial _l(a_2 \partial _m D_{t}r). \end{aligned}$$

The first and second terms on the RHS of the second equality have orders $\le 1/2$ and 1, respectively, so they produce terms of order at most $j-3/2$ when hit by $rD_{t}^{2j-3}$ and thus can be discarded. Continuing

$$\begin{aligned}{}[D_{t}, G^{lm}\partial _l (a_2 \partial _m \cdot ) ] r&\approx G^{lm} D_{t}\partial _l (a_2 \partial _m r) - G^{lm} \partial _l(a_2 \partial _m D_{t}r) \\&= G^{lm}\Big ( a_2 D_{t}\partial _l \partial _m r - a_2 \partial _l \partial _m D_{t}r \Big ) \\&\ \ \ + G^{lm} \Big ( D_{t}\partial _l a_2 \partial _m r + \partial _l a_2 D_{t}\partial _m r + D_{t}a_2 \partial _m \partial _l r - \partial _l a_2 \partial _m D_{t}r \Big ). \end{aligned}$$

All the terms inside the second parenthesis have orders at most 1 (thus giving order at most $j-3/2$ when hit by $rD_{t}^{2j-3}$) and can be discarded. The terms in the first parenthesis give $G^{lm} a_2 [D_{t}, \partial _l \partial _m]r$. Continuing

$$\begin{aligned}{}[D_{t}, G^{lm}\partial _l (a_2 \partial _m \cdot ) ] r&\approx G^{lm} a_2 [D_{t}, \partial _l \partial _m]r = G^{lm} a_2 \Big ( [D_{t},\partial _l] \partial _m r + \partial _l \left( [D_{t}, \partial _m] r \right) \Big ) \\&= G^{lm} a_2 \Big ( -\frac{a_0}{\kappa \langle r \rangle } G^{pq} \partial _l v_q \partial _p \partial _m r + \frac{\langle r \rangle ^{1+\frac{2}{\kappa }}}{(v^0)^3} v^p \partial _l r \partial _p \partial _m r \Big )\\&\quad \ + G^{lm} a_2 \partial _l \Big ( -\frac{a_0}{\kappa \langle r \rangle } G^{pq} \partial _m v_q \partial _p r + \frac{\langle r \rangle ^{1+\frac{2}{\kappa }}}{(v^0)^3} v^p \partial _m r \partial _p r \Big ). \end{aligned}$$

The second term in the first parenthesis has order 1. The second term in the second parenthesis produces, after differentiation by $\partial _l$, terms of order at most 1. Hence, the second terms in both parenthesis give order at most $j-3/2$ after we apply $rD_{t}^{2j-3}$ and belong to $F_{2j}$. Moreover, when $\partial _l$ in front of the second parenthesis hits the zero order coefficients in the first term it gives terms of order at most 1 which can again be discarded; when it hits $\partial _p r$ it produces a term that can be combined with the first term in the first parenthesis. Therefore, we have

5.15 $$\begin{aligned} D_{t}^{2j} r&\approx rD_{t}^{2j-3} \Big ( G^{ml} \partial _l (a_2 \partial _m D_{t}r ) \Big ) -r D_{t}^{2j-3} \Big ( \frac{a_0 a_2}{\kappa \langle r \rangle } G^{lm} \partial _l \partial _m v_q G^{pq} \partial _p r \Big ) \nonumber \\&\ \ \ -2 r D_{t}^{2j-3} \Big ( \frac{a_0 a_2}{\kappa \langle r \rangle } G^{lm} G^{pq} \partial _m v_q \partial _p \partial _l r \Big ). \end{aligned}$$

The last term on RHS (5.15) has a $\partial v \partial ^2 r$ factor. Hence it produces, after application of $r D_{t}^{2j-3}$ either non-endpoint terms or terms of order $< j-1$, so it belongs to $F_{2j}$.

We now analyze the second term on RHS (5.15). We distribute $D_{t}^{2j-3}$. Whenever at least one $D_{t}$ hits one of the zero order factors it results in a term of order $\le j-3/2$ that can be absorbed into $F_{2j}$. Hence we are left with

$$\begin{aligned}&-r \frac{a_0 a_2}{\kappa \langle r \rangle } G^{lm}G^{pq} D_{t}^{2j-3} \Big (\partial _l \partial _m v_q \partial _p r \Big ) \\&\quad = - r \frac{a_0 a_2}{\kappa \langle r \rangle } G^{lm}G^{pq} \sum _{\ell =0}^{2j-3} \left( {\begin{array}{c}2j-3\\ \ell \end{array}}\right) D_{t}^{2j-3-\ell } \partial _l \partial _m v_q D_{t}^\ell \partial _p r. \end{aligned}$$

The terms in the sum with $l \ne 0$ belong to $F_{2j}$. For, after commuting $D_{t}$ with $\partial$, we obtain either lower order terms or $\partial v \partial ^2 r$ factors, so we are left with

$$\begin{aligned} -r \frac{a_0 a_2}{\kappa \langle r \rangle } G^{lm}G^{pq} D_{t}^{2j-3} \partial _l \partial _m v_q \partial _p r&=\, -r \frac{a_0 a_2}{\kappa \langle r \rangle } G^{lm}G^{pq} \partial _l \partial _m D_{t}^{2j-3} v_q \partial _p r\\&\quad -r \frac{a_0 a_2}{\kappa \langle r \rangle } G^{lm}G^{pq} [D_{t}^{2j-3}, \partial _l \partial _m] v_q \partial _p r. \end{aligned}$$

The first term on the RHS belongs to $\tilde{L}_1 s_{sj-2}$. The second term on the RHS belongs to $F_{2j}$. This can be seen by computing the commutator in similar fashion to what we did to compute $[D_{t}, G^{lm}\partial _l (a_2 \partial _m \cdot ) ]$ (in fact, $[D_{t}^{2j-3}, G^{lm}\partial _l (a_2 \partial _m \cdot ) ] r$ and $[D_{t}^{2j-3}, \partial _l \partial _m]$ are the same modulo lower terms).

It remains to analyze the first term on RHS (5.15). We have

$$\begin{aligned} rD_{t}^{2j-3} \Big ( G^{ml} \partial _l (a_2 \partial _m D_{t}r ) \Big )&= G^{ml} \partial _l (a_2 \partial _m D_{t}^{2j-2} r ) + r [D_{t}^{2j-3}, G^{lm}\partial _l (a_2 \partial _m \cdot ) ] D_{t}r. \end{aligned}$$

The first term on the RHS belongs to $\tilde{L}_1 s_{2j-2}$. The term of order $j-1$ from the second term on the RHS is non-endpoint, as it comes from combining $\partial v$ from the commutator with $\partial v$ from $D_{t}r$.

We next consider the second term on (5.13). We have

5.16 $$\begin{aligned} - \frac{a_0}{\kappa \langle r \rangle } G^{lm} \partial _l r D_{t}^{2j-1} v_m&= - \frac{a_0}{\kappa \langle r \rangle } G^{lm} \partial _l r D_{t}^{2j-3} D_{t}(-a_2 \partial _m r)\nonumber \\&= \ \frac{a_0}{\kappa \langle r \rangle } G^{lm} \partial _l r D_{t}^{2j-3}\Big ( a_2 \partial _m D_{t}r + [D_{t}, a_2 \partial _m] r \Big ). \end{aligned}$$

Consider the second term on RHS (5.16). Using arguments similar to above, we can show that all terms belong to $F_{2j}$, except for the term that corresponds to all $D_{t}^{2j-3}$ hitting the $\partial v$ from the commutator $[D_{t},\partial _m]$, i.e., except for

$$\begin{aligned} - a_2\left( \frac{a_0}{\kappa \langle r \rangle } \right) ^2 G^{lm} \partial _l r G^{pq} D_{t}^{2j-3} \partial _m v_q \partial _p r&=- a_2\left( \frac{a_0}{\kappa \langle r \rangle } \right) ^2 G^{lm} \partial _l r G^{pq} \partial _m D_{t}^{2j-3} v_q \partial _p r\\&\quad - a_2 \left( \frac{a_0}{\kappa \langle r \rangle } \right) ^2 G^{lm} \partial _l r G^{pq} [D_{t}^{2j-3}, \partial _m] v_q \partial _p . \end{aligned}$$

The commutator term can again be shown to belong to $F_{2j}$ using the same sort of calculations as above. Modulo terms that can be absorbed into $F_{2j}$, the remaining term can be written as

$$\begin{aligned} a_2 \frac{a_0}{\kappa \langle r \rangle } G^{lm} \partial _l r\partial _m \Big ( -\frac{a_0}{\kappa \langle r \rangle } G^{pq} D_{t}^{2j-3} v_q \partial _p r \Big ) =&\, \frac{ a_2}{\kappa } G^{lm} \partial _l r\partial _m \Big ( -\frac{a_0}{\kappa \langle r \rangle } G^{pq} D_{t}^{2j-3} v_q \partial _p r \Big )\\&+ r a_3 G^{lm} \partial _l r\partial _m \Big ( -\frac{a_0}{\kappa \langle r \rangle } G^{pq} D_{t}^{2j-3} v_q \partial _p r \Big ), \end{aligned}$$

where we used (3.2). The first term on the RHS belongs to $\tilde{L}_1 s_{2j-2}$ and the second one can be absorbed into $F_{2j}$.

The first term on RHS (5.16) is treated with similar ideas. We notice that the top order term in that expression is

$$\begin{aligned}&\frac{a_0}{\kappa \langle r \rangle } G^{lm} \partial _l r a_2 \partial _m D_{t}^{2j-2} r =\, \frac{a_2}{\kappa } G^{lm} \partial _l r \partial _m D_{t}^{2j-2} r +ra_3 G^{lm} \partial _l r \partial _m D_{t}^{2j-2} r . \end{aligned}$$

The first term belongs to $\tilde{L}_1 s_{sj-2}$ and the second one to $F_{2j}$.

The case $j=2$ is done separately (since the definition of $s_2$ is different, recall (5.1)), but it follows essentially the same steps as above. Finally, the proof for $G_{2j}$ is done with the same type of calculations employed above and we omit it for the sake of brevity.

To continue our analysis, we need some coercivity estimates for the $\tilde{L}_1$, respectively $\tilde{L}_2+ \tilde{L}_3$. $\square$

#### Lemma 5.3

Assume that *A* is small. Then

5.17a $$\begin{aligned} \Vert s\Vert _{H^{2,\frac{1}{2\kappa } + \frac{1}{2}}}&\lesssim \Vert \tilde{L}_1 s\Vert _{H^{0,\frac{1}{2\kappa } - \frac{1}{2}}} +\Vert s\Vert _{L^2(r^\frac{1-\kappa }{\kappa })}, \end{aligned}$$

5.17b $$\begin{aligned} \Vert w\Vert _{H^{2,\frac{1}{2\kappa }+1}}&\lesssim \Vert (\tilde{L}_2+\tilde{L}_3)w\Vert _{H^{0,\frac{1}{2\kappa }} } + \Vert w\Vert _{L^2( r^\frac{1}{\kappa })}. \end{aligned}$$

Here we remark that the lower order terms on the right play no role in the proof, and can be omitted if (*s*, *w*) are assumed to have small support (by Poincare’s inequality), or if we use homogeneous norms on the left.

As a consequence of the second estimate above, we have

#### Corollary 5.4

Assume that *A* is small. Then

In Section 6 will also need the following straightforward alternative form of the above result:

#### Corollary 5.5

Assume that *B* is small. Then the same result as in Lemma 5.3 holds for the operators $L_1$, respectively $L_2+L_3$.

Here the smallness condition on *B* allows us to treat the differences $\tilde{L}_1 - L_1$, $\tilde{L}_2 - L_2$, $\tilde{L}_3 - L_3$ perturbatively.

#### Proof

We start with two simple observations. First of all, using a partition of unity one can localize the estimates to a small ball. We will assume this is done, and further we will consider the interesting case where this ball is around a boundary point $x_0$; the analysis is standard elliptic otherwise. We can assume that at $x_0$ on the boundary we have $\nabla r(x_0) = e_n$ so that in our small ball we have

5.18 $$\begin{aligned} |\nabla r - e_n| \lesssim A \ll 1. \end{aligned}$$

Secondly, the smallness condition on *A* guarantees that the coefficients *G* and $a_2$ have a small variation in a small ball, and we can freeze these coefficients modulo perturbative errors. Hence, we will simply freeze them, and assume that $a_2$ and *G* are constant. Then $a_2$ only plays a multiplicative role, and will be set to 1 for the rest of the argument.

A preliminary step in the proof is to observe that we have the weaker bounds

$$\begin{aligned} \Vert s\Vert _{H^{2,\frac{1}{2\kappa } + \frac{1}{2}}}&\lesssim \Vert \tilde{L}_1 s\Vert _{H^{0,\frac{1}{2\kappa } - \frac{1}{2}}} + \Vert s\Vert _{H^{1,\frac{1}{2\kappa } - \frac{1}{2}}}, \\ \Vert w\Vert _{H^{2,\frac{1}{2\kappa } + 1 }}&\lesssim \Vert (\tilde{L}_2 +\tilde{L}_3)w\Vert _{H^{0,\frac{1}{2\kappa } }} + \Vert w\Vert _{H^{1,\frac{1}{2\kappa }}}. \end{aligned}$$

These bounds can be proved in a standard elliptic fashion by integration by parts, e.g. in the case of the first bound one simply starts with the integral representing $\Vert \tilde{L}_1 s \Vert _{H^{0,\frac{1}{2\kappa }}}^2$ and exchange derivatives between the two factors. The details are left for the reader.

In view of the above bounds, it suffices to show that

5.19a $$\begin{aligned}&\Vert s\Vert _{H^{1,\frac{1}{2\kappa } -\frac{1}{2} }} \lesssim \Vert \tilde{L}_1 s\Vert _{H^{0,\frac{1}{2\kappa }- \frac{1}{2}} } +\Vert s\Vert _{L^2(r^\frac{1-\kappa }{2\kappa })}, \end{aligned}$$

5.19b $$\begin{aligned}&\Vert w\Vert _{H^{1,\frac{1}{2\kappa } }} \lesssim \Vert (\tilde{L}_2+\tilde{L}_3) w\Vert _{H^{0,\frac{1}{2\kappa } }} + \Vert w\Vert _{L^2( r^\frac{1}{\kappa })}. \end{aligned}$$

For (5.19a), compute

$$\begin{aligned} \int _{\Omega _t} r^{\frac{1-\kappa }{\kappa }} \partial _n s \tilde{L}_1 s \, dx&= \int _{\Omega _t} r^{\frac{1-\kappa }{\kappa }} \partial _n s G^{ij} a_2 \left( r \partial _i \partial _j s + \frac{1}{\kappa } \partial _i r \partial _j s \right) \, dx\\&= -\frac{1}{2} \int _{\Omega _t} r^\frac{1}{\kappa } a_2 \partial _n \left( G^{ij} \partial _i s \partial _j s\right) \, dx + \frac{1}{2} \int _{\Omega _t} r^\frac{1}{\kappa } a_2 \partial _n G^{ij} \partial _i s \partial _j s \, dx\\&\quad - \int _{\Omega _t} r^\frac{1}{\kappa } \partial _n s \partial _i(a_2 G^{ij}) \partial _j s \, dx\\&\gtrsim \int _{\Omega _t} r^\frac{1 - \kappa }{\kappa } a_2 G^{ij} \partial _i s \partial _j s \, dx + \int _{\Omega _t} r r^\frac{1 - \kappa }{\kappa } |\partial s|^2\, dx, \end{aligned}$$

which suffices, by the Cauchy-Schwarz inequality.

Now we consider (5.17b). As discussed above, we set $a_2=1$ and assume *G* is a constant matrix. We recall that $\tilde{L}_2$ has the form

5.20 $$\begin{aligned} (\tilde{L}_2 w)_i&= G^{ml} \left( \partial _i(r\partial _m w_l) + \frac{1}{\kappa } \partial _m r \partial _i w_l \right) \end{aligned}$$

while $\tilde{L}_3$ is given by

5.21 $$\begin{aligned} (\tilde{L}_3 w)_i&= r^{-\frac{1}{\kappa }} G^{ml} \partial _l \left( r^{1+\frac{1}{\kappa }} (\partial _m w_i - \partial _i w_m ) \right) \end{aligned}$$

Then a direct computation shows that

$$\begin{aligned} \begin{aligned} r^{\frac{1}{\kappa }}((\tilde{L}_2+ \tilde{L}_3) w)_i =&\ \partial _l ( G^{ml} r^{1+\frac{1}{\kappa }} \partial _m w_i) + r^\frac{1}{\kappa } (\partial _l r G^{lm} \partial _m w_i - \partial _l G^{lm} \partial _i w_m) \end{aligned} \end{aligned}$$

We will take advantage of the covariant nature of this operator in order to simplify it. Interpreting *G* as a dual metric and *w* as a one form, we see that the above operator viewed as a map from one forms to one forms is invariant with respect to linear changes of coordinates. Here we are interested in changes of coordinates which preserve the surfaces $x_n = const$. But even with this limitation, it is possible to choose a linear change of coordinates, namely the semigeodesic coordinates relative to the surface $x_n = 0$,

$$\begin{aligned} y' = Ax' + b x_n, \qquad y_n = x_n \end{aligned}$$

so that the metric *G* becomes a multiple of the identity. Then the estimate (5.19b) reduces to its euclidean counterpart, which is discussed in detail in [14] in the corresponding nonrelativistic context.

To finish the proof of Theorem 5.1, we will establish

5.22 $$\begin{aligned} \Vert (s_{2j-2},w_{2j-2})\Vert _{\mathcal {H}^{2k-2j+2}}&\lesssim \Vert (s_{2j},w_{2j})\Vert _{\mathcal {H}^{2k-2j}} + \varepsilon \Vert (r,v)\Vert _{\mathcal {H}^{2k}}, \, 1 \le j \le k, \end{aligned}$$

where $\varepsilon >0$ is sufficiently small. We are using $\varepsilon$ here to include two types of small error terms: (a) the terms that we estimate using *O*(*A*) as well as (b) the terms that have an extra factor of *r* and for which we can use smallness of *r* near the boundary; the latter type arise from the last term of (1.16a). Concatenating these estimates we then obtain the conclusion of the theorem.

To prove (5.22), we first consider $\Vert (F_{2j},G_{2j})\Vert _{\mathcal {H}^{2k-2j}}$. Using our interpolation inequalities, the non-endpoint property, and the structure of $(F_{2j},G_{2j})$ described in in Lemma 5.2, we obtain

$$\begin{aligned} \Vert (F_{2j},G_{2j})\Vert _{\mathcal {H}^{2k-2j}} \lesssim \varepsilon \Vert (r,v)\Vert _{\mathcal {H}^{2k}}. \end{aligned}$$

It remains to handle the term $\Vert (s_{2j},w_{2j})\Vert _{\mathcal {H}^{2k-2j}}$. For $j=k$ the desired estimate is a direct consequence of Lemma 5.3.

We move to treat the case $2 \le j < k$. The idea is to apply Lemma 5.3 with $s_{2j-2}$ and $w_{2j-2}$ replaced by suitable weighted derivatives of themselves. More precisely, we set

$$\begin{aligned} \left\{ \begin{aligned}&s {:=} L s_{2j-2}\\&w {:=} L w_{2j-2}, \end{aligned} \right. \end{aligned}$$

where

$$\begin{aligned} L = r^a \partial ^b, \quad 2a \le b \le 2(k-j). \end{aligned}$$

Applying *L* to (5.12a), we obtain

$$\begin{aligned} L s_{2j}&= \tilde{L}_1 L s_{2j-2} + [L,\tilde{L}_1 ] L s_{2j-2} + L F_{2j}. \end{aligned}$$

The term $L F_{2j}$ can again be dealt with using Lemma 5.2, as above. Thus we focus on the commutator. To analyze it, we consider induction on *a*, starting at $a=0$, and observe the following:

- All terms where at least one *r* factor gets differentiated twice are non-endpoint terms and can be estimated by interpolation.
- The terms where two *r* factors are differentiated are handled by the induction on *a*.
- Terms where only one *r* gets differentiated are also handled by induction on *a* unless $a=0.$

Therefore, all terms in the commutator where $a>0$ are perturbative terms. We now focus on the case $a=0$.

Consider a frame $(x^\prime , x^n)$ in Minkowski space that is adapted to a point near the boundary in the sense that

$$\begin{aligned} |\partial ^\prime r| \lesssim A, \quad |\partial _n r - 1 | \lesssim A. \end{aligned}$$

Then, all terms in the commutator with tangential derivatives only are error terms. For terms involving $\partial _n$, we find

$$\begin{aligned}{}[\partial _n^b,\tilde{L}_1] s&\approx b a_2 G^{ij} \partial _i \partial _j \partial _n^{b-1}s\\&\approx b a_2 G^{ij} \partial _i r \partial _j \partial _n^b s + b a_2 G^{n i^\prime } \partial _{i^\prime } \partial _n^b s + b a_2 G^{i^\prime j^\prime } \partial _{i^\prime } \partial _{j^\prime } \partial _n^{b-1}s, \end{aligned}$$

where primed indices run from 1 to $n-1$. The last two terms on the RHS can be treated by yet another induction, this time over *b*. The first term on the RHS can be combined back with $\tilde{L}_1$, yielding $\partial _n^b \tilde{L}_1 \approx \tilde{L}^b_1 \partial _n^b$, where

$$\begin{aligned} \tilde{L}_1^b = r a_2 G^{ij} \partial _i \partial _j s + a_2 \left( \frac{1}{\kappa } + b \right) G^{ij} \partial _i r \partial _j s. \end{aligned}$$

The operator $\tilde{L}_1^b$ has a similar structure to $\tilde{L}_1$, and an inspection in the proof of Lemma 5.3 shows that the corresponding coercive estimate for *s* holds with $\tilde{L}_1^b$ in place of $\tilde{L}_1$.

The above argument works for $j\ge 2$ in that (5.12a) is valid only for $j\ge 2$. However, a minor change in the above using the definition $s_2$ yields the result also for $j=1$. This takes care of the *s* terms in (5.22); the proof for the *w* terms is similar. $\square$

### Energy estimates

In this Section we establish

#### Theorem 5.6

The energy functional $E^{2k}$ defined in (5.7) satisfies the following estimate:

$$\begin{aligned} \frac{d}{dt} E^{2k}(r,v) \lesssim _A B \Vert (r,v) \Vert _{\mathcal {H}^{2k}}^2. \end{aligned}$$

#### Proof

In view of Eqs. (5.2a)–(5.2b) and (5.5), the the energy estimates for the linearized equation in Section 3, and estimates for transport equations, it suffices to show that the terms $f_{2k}$, $g_{2k}$ and $h_{2k}$, given by (5.3a), (5.3b), and (5.6), respectively, are perturbative, i.e., they satisfy the estimate

$$\begin{aligned} \Vert (f_{2k}, g_{2k} ) \Vert _{\mathcal {H}} + \Vert h_{2k}\Vert _{L^2( r^\frac{1}{\kappa })} \lesssim B \Vert (r,v)\Vert _{\mathcal {H}^{2k}}. \end{aligned}$$

To prove this bound we need to understand the structure of $(f_{2k},g_{2k})$, respectively $h_{2k}$. $\square$

#### Lemma 5.7

Let $k \ge 1$. Then source terms $f_{2k}$ and $g_{2k}$ in the linearized Eq. (5.2) for $(s_{2k},w_{2k})$, given by (5.3a)-(5.3b) are multilinear expressions in $(r,\nabla v)$, with coefficients which are smooth functions of (*r*, *v*), which have order $\le k-\frac{1}{2}$, respectively $\le k$, with exactly $2k+1$ derivatives, and which are not endpoint, in the sense that there is no single factor in $f_{2k}$, respectively $g_{2k}$ which has order larger that $k-1$, respectively $k-\frac{1}{2}$.

Similarly, the source term $h_{2k}$ in the vorticity transport Eq. (5.5), given by (5.6), has the same properties as $g_{2k}$ above.

Once the lemma is proved, arguing similarly to Section 5.2, we see that this suffices to apply our interpolation results in Propositions 2.6, 2.9 and 2.10 and obtain the desired bound. Here we remark that a scaling analysis shows that in the interpolation estimates we need to use at most one *B* control norm, with equality exactly in the case of terms of highest order. One should also compare with the situation in the similar computation in [14], where no lower order terms appear. Hence, the poof of the theorem is concluded once we prove the above lemma.

#### *Proof of Lemma *5.7

Consider first $f_{2k}$. The fact that all terms in $f_k$ have order at most $k-\frac{1}{2}$ is obvious. The non-endpoint property can be understood as asking that there are no derivatives of order $2k+1$, and that, in addition, for the terms of maximum order, they have at least two factors of the form $\partial ^{2+} r$ or $\partial ^{1+}v$. Notably, this excludes any terms of the form

$$\begin{aligned} f(r,v) r^{k+1-j}(\nabla r)^j \partial ^{2k+1-j} v, \qquad 0 \le j \le k+1. \end{aligned}$$

A similar reasoning applies for $g_{2k}$ and $h_{2k}$, where the forbidden terms are those with a factor with $2k+1$ derivatives, as well as those of maximum order of the form

$$\begin{aligned} f(r,v) r^{k-j}(\nabla r)^j \partial ^{2k+1-j} r, \qquad 0 \le j \le k. \end{aligned}$$

We start with a simple observation, which is that, if in (5.3a) or (5.3b), any derivative falls on a coefficient such as $G$, $a_0$, $a_1$, or $a_2$, then we obtain lower order terms which automatically satisfy the above criteria. Thus, for the purpose of this Lemma we can treat these coefficients as constants.

A second observation is that there are no factors with $2k+1$ derivatives in either $s_{2k}$ or $w_{2k}$, due to the commutator structures present in both (5.3a) or (5.3b). This directly allows us to discard all lower order terms, and in particular those containing $a_1$ and $a_3$. By the same token we can set $a_0=1$ and $\langle r \rangle = 1$.

Given the above observations, it suffices to consider the reduced expressions

5.23 $$\begin{aligned} f_{2k}^{reduced}&= \ G^{ij} [ r \partial _i, D_{t}^{2k} ] v_j -\frac{1}{\kappa } G^{ij} D_{t}\left( \partial _i r \right) D_{t}^{2k-1} v_j \end{aligned}$$

5.24 $$\begin{aligned} (g_{2k}^{reduced})_i&= \ a_2( D_{t}^{2k-1} [ \partial _i, D_{t}] r - \frac{a_0}{\kappa \langle r \rangle }G^{jl} \partial _j r \partial _i D_{t}^{2k-1} v_l)\nonumber \\&\quad + a_2( [\partial _i, D_{t}^{2k-1} ] D_{t}r - \frac{1}{\kappa } G^{ml} \partial _i \partial _m r D_{t}^{2k-1} v_l), \end{aligned}$$

Consider $f_{2k}^{reduced}$ first. When commuting $\partial$ and $D_{t}^{2k}$, this produces at least one $\partial v$, so $[r \partial _i, D_{t}^{2k} ] v_j$ is not an endpoint term. Similarly, $D_{t}\left( \partial _i r \right)$ has order 1/2 so the second expression is also not an endpoint term.

We now investigate $g_{2k}^{reduced}$. Neither of the first two terms is perturbative, but we have a leading order cancellation between them, based on the relations

$$\begin{aligned}{}[D_{t}, \partial _i]= - \partial _i \left( \frac{v^j}{v^0} \right) \partial _j, \end{aligned}$$

and

5.25 $$\begin{aligned} \partial _i \left( \frac{v^j}{v^0} \right) = \frac{a_0}{\kappa \langle r \rangle } G^{jl} \partial _i v_l - \frac{\langle r \rangle ^{1+\frac{2}{\kappa }}}{(v^0)^3} v^j \partial _i r. \end{aligned}$$

The contribution of the second term is lower order and thus perturbative. The contribution of the first term is combined with the second term in (5.24) to obtain a commutator structure

$$\begin{aligned} \left[ D_{t}^{2k-1}, \frac{a_0}{\kappa \langle r \rangle } G^{jl} \partial _j r \partial _i \right] v_l, \end{aligned}$$

which yields only balanced terms.

The third term in (5.24) is also balanced due to the commutator structure, while the last term has a direct good factorization.

We next move to $h_{2k}$. From (5.6) we see that we are commuting $r^a \partial ^b$ with either $D_{t}$ or $\partial v$, so we always obtain $\partial v$ factors that give non-endpoint terms. The only possible exception is when all derivatives in the commutator with $D_{t}$ are applied to the *r* term in $v^0$. But this yields a lower order term.$\square$

## Construction of regular solutions

In this section we provide the first step in our proof of local well-posedness, namely, here we present a constructive proof of regular solutions. The rough solutions are obtained in the last section as unique limits of regular solutions.

Given an initial data $(\mathring{r},\mathring{v})$ with regularity

$$\begin{aligned} ( \mathring{r},\mathring{v}) \in {\mathbf{H} }^{2k}, \end{aligned}$$

where *k* is assumed to be sufficiently large, we will construct a local in time solution, bounded in ${\mathbf{H} }^{2k}$, with a lifespan depending on the ${\mathbf{H} }^{2k}$ size of the data.

### Construction of approximate solutions

We discretize the problem with a time-step $\epsilon > 0$. Then, given an initial data $( \mathring{r},\mathring{v}) \in {\mathbf{H} }^{2k}$, our objective is to produce a discrete approximate solution $(r(j\epsilon ), v(j\epsilon ))$, with properties as follows:

- (Norm bound) We have
- (Approximate solution)

The first property will ensure a uniform energy bound for our sequence. The second property will guarantee that in the limit we obtain an exact solution. There we use a weaker topology, where the exact choice of norms is not so important (e.g. $C^2$).

Having such a sequence of approximate solutions, it is straightforward to produce, as the limit on a subsequence, an exact solution (*r*, *v*) on a short time interval which stays bounded in the above topology. The key point is the construction of the above sequence. It suffices to carry out a single step:

#### Theorem 6.1

Let *k* be a large enough integer. Let $(\mathring{r},\mathring{v}) \in {\mathbf{H} }^{2k}$ with size

$$\begin{aligned} E^{2k}(\mathring{r},\mathring{v}) \le M, \end{aligned}$$

and $\epsilon \ll _M 1$. Then there exists a one step iterate  with the following properties:

1. (Norm bound) We have
2. (Approximate solution)  where $\mathring{G}$, $\mathring{a}_1$, and $\mathring{a}_2$ are $G$, $a_1$, and $a_2$ evaluated at $(\mathring{r},\mathring{v})$.

The strategy for the proof of the theorem is the same as in the last two authors’ previous paper [14], by splitting the time step into three:

- Regularization,
- Transport,
- Euler’s method,

where the role of the first two steps is to improve the error estimate in the third step. The regularization step is summarized in the next Proposition:

#### Proposition 6.2

Given $(\mathring{r},\mathring{v}) \in {\mathbf{H} }^{2k}$, there exist regularized versions (*r*, *v*) with the following properties:

$$\begin{aligned} r-\mathring{r}= O(\epsilon ^{2}), \qquad v - \mathring{v}= O(\epsilon ^{2}), \end{aligned}$$

respectively

$$\begin{aligned} E^{2k} (r,v) \le (1+ C \epsilon ) E^{2k} (\mathring{r},\mathring{v}) , \end{aligned}$$

and

$$\begin{aligned} \Vert (r,v)\Vert _{{\mathcal H }^{2k+2}} \lesssim \epsilon ^{-1}M. \end{aligned}$$

#### Proof

We repeat the construction in [14]. There are only a few minor differences, namely

- The self-adjoint operators $L_1$, $L_2$ and $L_3$ there are replaced by their counterparts in this paper, i.e., (3.7a), (3.10), and (3.9) (recall that $L_1=\hat{L}_1$ and $L_3 = \tilde{L}_3$).
- Using (3.12), relations similar to (5.12) continue to hold for the self-adjoint operators. Thus, the approximate relations between $(s_{2k},w_{2k})$ and $(s^-_{2k},w^-_{2k})$ in Section 6 of [14] also hold here.
- The elliptic estimates of Lemma 5.3 hold for $L_1$ and $L_2,L_3$, with essentially the same proof.

Aside from the above minor differences, the most important observation in invoking the proof given in [14] is that the counterpart of Lemma 6.3 in [14] still holds with a minor change. For convenience we state here its counterpart (below, $D s_{2k}$ and $D w_{2k}$ are the differentials of $s_{2k}$ and $w_{2k}$ as functions of *r* and *v*):

#### Lemma 6.3

We have the algebraic relationswhere the error terms $(\tilde{F}_{2k},\tilde{G}_{2k})$ are linear in ,Their coefficients are multilinear differential expressions in , have order at most $k-1$, respectively $k-\frac{1}{2}$, and whose monomials fall into one of the following two classes:

i) Have maximal order but contain at least one factor with order $> 0$, i.e.  or , or
ii) Have order strictly below maximum.

By comparison, the similar relations in Lemma 6.3 in [14] are homogeneous, so only terms of type (i) arise in the error terms. Here our equations are no longer homogeneous, and lower order terms do appear. In particular, we note that all the contributions coming from the last term in the first equation (1.16a) belong to the class (ii) above. This is correlated with and motivates the fact that this term was neglected in our definition of the operator $L_1$.

With these observations in mind, the proof given in [14] applies directly here. We now use Proposition 6.2 in order to prove Theorem 6.1.

*Proof of Theorem* 6.1. For the transport step, we definewhere, in agreement with the our definition of the material derivative, we iterate the coordinates by flowing with $v^i/v^0$, and not simply $v^i$.

Then we carry out the Euler step, and define $(r^0,v^0)$ byTo show that  have the properties in the Theorem, the argument is completely identical to the one in [14]. $\square$

## Rough solutions and continuous dependence

The last task of the current work is to construct rough solutions as limits of smooth solutions, and conclude the proof of Theorem 1.2. Fortunately, the arguments in the preceding paper [14] by the last two authors for the similar part of the results apply word for word. This is despite the fact there are several differences between the two problems that play a role on how the energy estimates are obtained, as well as on how uniqueness is proved. However, the functional framework developed in [14] and also implemented here does not see these differences. Furthermore, the proof of the similar result in [14] only uses (i) the regularization procedure in Section 2, (ii) the difference bounds of Theorem 1.1, and (iii) the energy estimates of Theorem  1.3, without any reference to their proof.

Thus, in our current result we rely on the same succession of steps as in the non-relativistic companion work of the last two authors [14], which we briefly outline here for the reader. These steps are

*1. Regularization of the initial data.* We regularize the initial data; this is achieved by considering a family of dyadic regularizations of the initial data as described in Section 2. These data generate corresponding smooth solutions by Theorem 1.2. For these smooth solutions we control on the one hand higher Sobolev norms $\mathcal {H}^{2k+2j}$ using our energy estimates in Theorem 1.3, and on the other hand the $L^2$-type distance between consecutive solutions, which is at the level of the $\mathcal {H}$ norms, by Theorem 1.1.

*2. Uniform bounds for the regularized solutions.* To prove these bounds we use a bootstrap argument on our control norm *B*, where *B* is time dependent. The need for an argument of this kind is obvious. Once we have the regularized data sets $(\mathring{r}^h,\mathring{v}^h)$, we also have the corresponding smooth solutions $(r^h,v^h)$ generated by the smooth data $(\mathring{r}^h,\mathring{v}^h)$. A-priori these solutions exist on a time interval that depends on *h*. Instead, we would like to have a lifespan bound which is independent of *h*. This step requires closing the bootstrap argument via the energy estimates already obtained in Section 5.

*3. Convergence of the regularized solutions.* We obtain the convergence of the regular solutions $$(r^h,v^h)$$ to the rough solution (*r*, *v*) by combining the high and the low regularity bounds directly. This yields rapid convergence in all $$\mathbf {H}^{2k'}$$ spaces below the desired threshold, i.e. for $$k'<k $$. Here we rely primarily on results in Section 4, namely Theorem 1.1.

*4. Strong convergence.* Here we prove the convergence of the smooth solutions to the rough limit in the strong topology ${\mathbf{H} }^{2k}$. To gain strong convergence in ${\mathbf{H} }^{2k}$ we use frequency envelopes to more accurately control both the low and the high Sobolev norms above. This allows us to bound differences in the strong ${\mathbf{H} }^{2k}$ topology. A similar argument yields continuous dependence of the solutions in terms of the initial data, also in the strong topology. For more details we refer the reader to [14].

## Footnotes

1 Observe that the requirement $0\le c_s^2 < 1$ imposes a bound on $\varrho$. This occurs because power-law equations of state such as (1.5) are no longer valid if the density is very large [17].

2 In an appropriately weighted sense in the case of $\varrho$, see Theorem 1.1.

3 While this problem does not have an exact scaling symmetry, one can still identify a leading order scaling.

4 It is well known that we can think of the relativistic Euler flow as a wave-transport system. What is relevant here is that the wave evolution that comes out of the diagonalized equations allows estimates all the way to the free surface.

5 The factor $\frac{1}{\kappa }$ in the definition of *r* is a matter of convenience. Although *r* and $c_s^2$ differ by this factor, we slightly abuse the terminology and also call *r* the sound speed squared.

6 And then indeed turns out to be lower order from a scaling perspective.

7 In [14] the *A* component is omitted, and *B* is a homogeneous norm. But here, we need to also add the lower order component *A* in order to be able to handle lower order terms.

8 In order to provide some context, we briefly discuss the general relativistic free-boundary Euler equations, i.e., including both the cases of a gas and a liquid. We do not, however, make an overview of related works that treat the non-relativistic free-boundary Euler equations. See [14] and references therein for such a discussion.

9 More precisely, only a type of partial uniqueness has been obtained, see the discussion in [35].

10 Heuristically, both would be fulfilled by an appropriate Weyl type paradifferential quantization, but that would be very cumbersome to use in the presence of the free boundary.
